# SRC Homology 2 Domain Binding Sites in Insulin, IGF-1 and FGF receptor mediated signaling networks reveal an extensive potential interactome

**DOI:** 10.1186/1478-811X-10-27

**Published:** 2012-09-14

**Authors:** Bernard A Liu, Brett W Engelmann, Karl Jablonowski, Katherine Higginbotham, Andrew B Stergachis, Piers D Nash

**Affiliations:** 1 Ben May Department for Cancer Research, The University of Chicago, Chicago, IL, 60637, USA; 2 Committee on Cancer Biology, The University of Chicago, Chicago, IL, 60637, USA; 3 Department of Biochemistry and Molecular Biology, The University of Chicago, Chicago, IL, 60637, USA; 4 Samuel Lunenfeld Research Institute, Mount Sinai Hospital, Toronto, ON, Canada; 5 Department of Biomedical Informatics and Medical Education, University of Washington, Seattle, WA, USA

## Abstract

Specific peptide ligand recognition by modular interaction domains is essential for the fidelity of information flow through the signal transduction networks that control cell behavior in response to extrinsic and intrinsic stimuli. Src homology 2 (SH2) domains recognize distinct phosphotyrosine peptide motifs, but the specific sites that are phosphorylated and the complement of available SH2 domains varies considerably in individual cell types. Such differences are the basis for a wide range of available protein interaction microstates from which signaling can evolve in highly divergent ways. This underlying complexity suggests the need to broadly map the signaling potential of systems as a prerequisite for understanding signaling in specific cell types as well as various pathologies that involve signal transduction such as cancer, developmental defects and metabolic disorders. This report describes interactions between SH2 domains and potential binding partners that comprise initial signaling downstream of activated fibroblast growth factor (FGF), insulin (Ins), and insulin-like growth factor-1 (IGF-1) receptors. A panel of 50 SH2 domains screened against a set of 192 phosphotyrosine peptides defines an extensive potential interactome while demonstrating the selectivity of individual SH2 domains. The interactions described confirm virtually all previously reported associations while describing a large set of potential novel interactions that imply additional complexity in the signaling networks initiated from activated receptors. This study of pTyr ligand binding by SH2 domains provides valuable insight into the selectivity that underpins complex signaling networks that are assembled using modular protein interaction domains.

## Lay abstract

Every cell in our body is an immensely powerful computational device capable of integrating vast amounts of data from intrinsic and extrinsic cues and responding with remarkable fidelity. What underlines this computational power are not static wires, but dynamic interactions that leverage the finite number of genes to generate an almost infinite number of combinatorial interactions between protein components. In the post-genomics era, mapping these interactions represents a next frontier. The sum total of all permitted interactions is referred to as the potential interactome. In any given cell, only a subset of potential interactions will be enabled and this defines the selective differences in signalling between tissues. Understanding the whole provides insight into the information processing power of the system and may suggest new avenues for therapeutic intervention to treat diseases caused by faults in signal processing mechanisms. This study outlines the potential interactome for initial signalling events from the insulin receptor, insulin-like growth factor receptor and all four members of the fibroblast growth factor receptor family. These systems are essential for human development and dysfunctional signalling has been implicated in a wide range of human diseases including diabetes, many cancers, Alzheimer's disease, many developmental disorders and even aging. Binary connections are reported between 50 SH2 domain-containing proteins and 192 phosphopeptide nodes on 13 signal-initiating proteins. This verified almost every interaction described in the past 25 years and adds an extensive new data, providing a step towards fathoming the intricacies of differential cell communication between various tissues and disease states.

## Introduction

Signaling immediately downstream of receptor tyrosine kinases (RTKs) is accomplished in large part by the recruitment of phosphotyrosine (pTyr) interacting proteins to sites of tyrosine phosphorylation on the activated receptors and their associated scaffold proteins [1-3]. A given RTK may contain on the order of 10–20 phosphorylatable tyrosine residues with additional sites available on associated scaffold proteins resulting in a large number of potential sites for recruiting binding partners. The majority of phosphotyrosine interacting proteins contain a conserved Src homology 2 (SH2) domain [4]. The SH2 domain is the classic archetype for the large family of modular protein interaction domains that serve to organize a diverse array of cellular processes [5,6]. SH2 domains interact with phosphorylated tyrosine-containing peptide sequences [7-11] and in doing so they couple activated protein tyrosine kinases (PTKs) to intracellular pathways that regulate many aspects of cellular communication in metazoans [12,13]. The human genome encodes 111 SH2 domain proteins [14,15] that represent the primary mechanism for cellular signal transduction immediately downstream of PTKs. As one might expect, SH2 domain proteins play an essential role in development and have been linked to a wide array of human malignancies including cancers, diabetes, and immunedeficiencies [14,16].

Despite the importance of SH2-mediated signaling in human disease, our understanding of their interactions remains far from complete. Direct experimental measurement of binding partners has typically focused on specific interactions driven by hypotheses relating to the precise signaling events under investigation. This yields a set of high quality, but inevitably sparse data. Certain pTyr proteins and SH2 domains are extensively studied while others are more arcane. Nonetheless, the SH2-mediated interactions reported over 25 years of intensive study provide a solid foundation for validating high-throughput datasets.

SH2 domain interactions are almost always phosphorylation dependent as roughly half of the binding energy is devoted to pTyr recognition [17,18]. Despite this, SH2 domains preserve substantial specificity for peptide ligands, recognizing residues adjacent to the pTyr, particularly those at positions +1 to +5 C-terminal to the critical pTyr [19-21]. This is achieved in part by use of complex recognition events that effectively combine the use of motifs and sub-motif modifiers [11]. Specifically, SH2 domains recognize targets not only through permissive residues adjacent to the phosphotyrosine that constitute binding motifs, but also by making use of contextual sequence information and non-permissive residues [22] to define highly selective interactions with physiological peptide ligands. The specificity of SH2 domains enables their use as tools to profile the global phosphotyrosine state of cells or tissues [23-27], without *a priori* knowledge of the specific target proteins or peptides. Profiling signaling using SH2 domains has direct implications to diagnosis and guiding therapeutic decisions as the patterns obtained can be used to classify tumors [27]. The ligand specificity of many SH2 domains has been evaluated using approaches including synthetic peptide libraries [19,28,29], oriented peptide libraries [20,30] and phage display [31]. Information of this type is often described by position-specific scoring matrices (PSSM), and allows programs such as ScanSite and Scoring Matrix-Assisted Ligand Identification (SMALI) to predict potential binding motifs [20,21].

Recruitment of SH2 domain proteins to phosphorylated sites is a dynamic process and is by no means predetermined by the phosphorylation event alone. Each tyrosine site on a scaffold (including sites on receptors that recruit SH2 domains) can be phosphorylated or unphosphorylated. The phosphorylated site can either be free or occupied by one of its potential binding partners. Each possible assembly of interaction partners on a given scaffold represents an interaction microstate [32-35]. The actual populated interaction microstates from which signaling develops is a function of many factors, including protein expression levels, local concentration, and the probability that a given site is phosphorylated. Thus, distinct signaling networks may originate from the same scaffold or receptor in different cell types. This is also true under conditions of aberrant expression of signaling components that are a common occurrence in pathologies such as cancer. Thus, accurate and well-annotated potential interactomes that represent the aggregate available interaction microstates are a valuable resource that opens the door to interpreting studies of signaling in different cell types or under conditions of altered protein expression. As the Human Protein Atlas detailing subcellular localization data and expression data makes clear, cell lines and tissues vary widely and often in unanticipated ways in terms of protein expression [36]. All of this suggests that detailed potential interactomes may provide substantial benefit in understanding cell-type specific signaling.

Herein, we describe a potential interactome obtained using addressable peptide arrays consisting of 192 physiological peptides from the insulin (Ins), insulin growth factor 1 (IGF-1) and fibroblast growth factor (FGF) signaling pathways to identify interactions with 50 SH2 domains. This set represents a broad sampling of the SH2 domains extant in the human genome. The results of this study map a range of potential phosphotyrosine-dependent interactions within the FGF and Ins/IGF-1 pathways. These signaling systems have relevance to understanding complex multi-tissue pathologies such as diabetes and cancer as well as in normal physiology and development. This study confirms 44 of 54 previously described interactions. In addition, we report an extensive set of novel interactions. Validation of 60 binary interaction pairs was conducted using the orthogonal method of solution binding measured by fluorescence polarization. The binding motifs obtained for each SH2 domain closely match those reported in a number of independent studies. Protein co-precipitation experiments, or endogenous phosphorylation upon receptor stimulation, were further used to validate a number of interactions. The results of this study highlight the available pool of potential SH2-mediated interactions with these 13 major signaling proteins and serve as a first step in understanding signaling microstate variations. Interactive figures and additional information may be found at http://www.sh2domain.org.

## Results

### Peptide arrays for SH2 interactions within the FGF/Ins/IGF-1 signaling pathways

**Figure 1:**
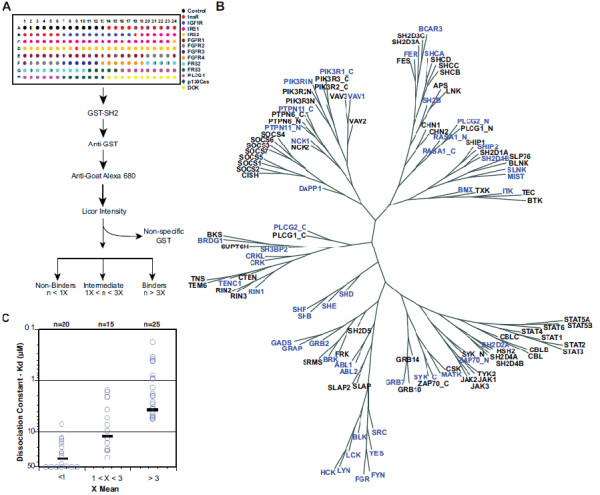
**Probing interactions between SH2 domains and physiological peptide ligands at a systems level. **(**A**) A representation of a SPOT peptide array containing 192 phosphotyrosine peptides including control peptides (black) and peptides from the 13 proteins present on the array indicated by their represented colors. SPOT peptide arrays were incubated with 250nM GST-SH2 domain as indicated. Interactions were detected using anti-GST antisera and Alexa-680-labeled anti-mouse secondary antibody and the intensity of signals recorded using LiCor Odyssey. (**B**) Neighbor-Joining Tree of all 121 SH2 domains. Highlighted in blue are the 50 SH2 domains selected across different families for this study. (**C**) Peptide arrays using SPOTS is a semi-quantitative method for measuring protein domain-pTyr peptide interactions. The dissociation constants (K_D_) were measured between 60 interaction pairs presenting interactions determined using peptide arrays as greater than 3X the mean, between 1 and 3X the mean and less than 3X the mean. The mean K_D_ value for each group is marked with a black line.

The use of addressable peptide arrays is a reproducible and semi-quantitative approach that has been extensively validated for studying protein interactions with peptide ligands [37-39]. To investigate connections between SH2 domain proteins and their putative phosphorylated docking sites on cell surface receptors, we developed addressable arrays consisting of 192 phosphotyrosine peptides. This peptide set was assembled using 71 phosphotyrosine peptide motifs corresponding to all of the cytoplasmic tyrosine residues within the FGF receptors (FGFR1-4), insulin receptor (InsR) and IGF-1 receptor (IGF-1R) (Figure 1A). Activation of these receptors results in the phosphorylation of associated scaffold proteins, and so 75 phosphotyrosine peptides corresponding to a comprehensive list of tyrosine residues within insulin receptor substrates (IRS-1 and IRS-2) and fibroblast receptor substrates (FRS-2 and FRS-3) were included. In addition, 33 phosphotyrosine peptides were incorporated from the downstream signaling proteins PLC-γ1, p130Cas (BCAR1) and p62DOK1. Finally, a set of 12 positive control peptides corresponding to 19 reported interactions with 15 SH2 domains for which equilibrium dissociation constant (K_D_) values span a range from low nM to 50 μM were incorporated to aid in validating the results. These control peptides provide a reference and establish the empirical cut-off for designated binding interactions (Table 1). No discrimination was made against peptides on the basis of reported phosphorylation state in order to examine a diverse and unbiased set of motifs. The resulting set of 192 phosphotyrosine peptides and their corresponding position in the proteins of origin is noted in Additional file 1: Table S1. Addressable arrays were synthesized as membrane-bound 11-mer peptides using the SPOT synthesis technique [40-42]. While the majority of SH2 domains recognize residues C-terminal to the phosphotyrosine in their cognate peptide ligands, additional contacts between SH2 domains and residues N-terminal to the phosphotyrosine are observed for the SH2 domain of Sh2d1a (SAP) [43] and cannot be ruled out in other cases. Peptides were synthesized with six flanking residues C-terminal to the phosphotyrosine and four residues N-terminal to the phosphotyrosine.

**Table 1:** **Literature confirmed interactions 39 array-positive interactions were experimentally verified or confirm previously reported interactions while 23 array-negative interactions empirically suggest a threshold corresponding to a K**_**D**_**of approximately 5 to 10 μM for this data set**

**Peptide sequence**	**Protein/position**	**Expected partner**	**Array positive**	**Affinity (K**_**D**_**)/relative affinity (IC**_**50**_**)**	**References**	**Comments and other SH2 domains bound**
ATDDpYAVPPPR	p62DOK1 (Y409), similar to p130Cas (Y410)	Crk	Yes	N.A.	[87]	Crkl, Hck
EDDGpYDVPKPP	Cbl (Y774)	Crk	Yes	N.A.	[88]	Crkl
		Hck	Yes	N.A.	[89,90]	Fgr, Src, Yes
AEDVpYDVPPPA	p130Cas (Y362)	Crk	Yes	K_D_ = 0.545 μM	[87]	Src, Hck, Fyn
GLDEpYDEVPMP	B3AT (Y921)*	Nck1	Yes	K_D_ = 0.06 μM	[91]	*Similar to the TIR10 peptide (EHIpYDEVAAD). Fer
DDPSpYVNVQNL	ShcA (Y426)	Grb2	Yes	K_D_ = 23nM	[92,93]	Gads, Grap
		Grb2	Yes	K_D_ = 53 ± 8nM	[94]	
ADNDpYIIPLPD	PDGFRb (Y1021)	Plcγ-N	Yes	K_D_ = 0.65 - 2.2nM^1^	[95]	^1^Tandem SH2 domains of PLCG1 were tested against a tandem phospho-peptide of PDGFRβ Y1009/Y1021.
		Plcγ-C	Yes	K_D_ = 0.65 - 2.2nM^1^	[95-97];	Brk
		Plcγ-C	Yes	K_D_ = 4.1 ± 0.8 μM	[98]	
		Vav1	Yes	N.A.	[99]	
		PI3K_N	No	ID_50_ = 45 ± 14 μM	[79]	Below Threshold
SLTIpYAQVQKA	SLAM (Y280)	Sh2d1b	Yes	K_D_ = 131nM	[100]	
HDGLpYQGLSTA	CD150 (Y142)	Shc1	No	K_D_ = 50 μM	[101]	Below Threshold
STVEpYSTVVHS	gp130/IL-6 Receptor (Y759)	Ptpn11	No	N.A.	[102]	PTPN11_N is 1.33X Mean, PTPN11_C is 2.8X Mean Brdg1, Brk, Sh2d1b
AEPQpYEEIPIY	Middle T-antigen (Y323)	Src	Yes	K_D_ = 0.55-0.8 μM	[19]	Blk, Brk, Fer, Fgr, Hck, Lyn, Nck1, Shc1, Yes
		Lck	Yes	K_D_ = 1.3 ± 0.2 μM	[103]	
SDDDpYDDVDIP	HPK1 (Y379)	Nck1	Yes	N.A.	[104]	Fer, SLNK (similar to BLNK and SLP76)
TRDIpYETDpYpYR	InsR (Y1185,89,90)	Plcγ_C	Yes	N.A.	[105]	Crk, Crkl, Fer, Grb7, PI3K1_C
EDLSpYGDVPPG	IRS-1 (Y151)	Nck1	No	N.A.	[106]	1.76X Mean Abl1, Blk, Fyn, Lck, Lyn, Sh2d1b, Shc1, Ship2, Slnk, Yes
ELSNpYICMGGK	IRS-1 (Y465)	Ptpn11_N	No	IC_50_ = 48 ± 16 μM	[107]	Below Threshold, 0.9X Mean
SIEEpYTEMMPA	IRS-1 (Y551)	Ptpn11_N	No	IC_50_ = 11 ± 1.0 μM	[107]	Below Threshold, 0.56X Mean
GSGDpYMPMSPK^2^	IRS-1 (Y612)	PI3K1_N	No	ID_50_ = 0.7-1.1 μM	[79]	^2^Peptide Y632 (GSGDpYMPMSPK) on IRS-1 is similar to Y612 (TDDGpYMPMSPG) on IRS-1.
DPNGpYMMMSPS	IRS-1 (Y662)	Ptpn11_N	No	IC_50_ = 96 ± 13 μM	[107]	Below Threshold, 0.54X Mean
SPGEpYVNIEFG	IRS-1 (Y896)	Ptpn11_N	Yes	IC_50_ = 4.8 ± 1.0 μM	[107,108]	Abl2, Blk, Dapp1, Grb7, Itk, Mist, PI3K1_N, PI3K1_C, PTPN11_N, PLCγ_C, Rasa1_N, Rasa1_C, Sh2b, Sh2d1b, Shb, Shf, Shd, She, Syk_C, Vav1, Yes
		Grb2	Yes	K_D_ = 35nM	[92,108,109]	Gads, Grap
APVSpYADMRTG	IRS-1 (Y1012)	Ptpn11_N	No	K_D_ = 110 ± 23 μM	[107]	Below Threshold, 0.59X Mean
NGLNpYIDLDLV	IRS-1 (Y1179)	Ptpn11_N	Yes^3^	K_D_ = 3.0 ± 0.60nM	[95,108,110]	^3^Tandem SH2 domains of PTPN11 was used to bind to the tandem motif of IRS-1
		Ptpn11_N	Yes	IC_50_ = 1.1 ± 0.5 μM	[107]	Abl1, Ptpn11_N, Plcγ_C, Rasa1_N, Shb, Shf, Shd, She, Yes
		Fyn	No	N.A.	[46]	0.45X Mean
DLSApYASISFQ	IRS-1 (Y1229)	Ptpn11_N	No	IC_50_ = 25 ± 4.2 μM	[107]	Below Threshold, 2.78X Mean
		Ptpn11_C	No	N.A.	[108]	1.59X Mean
		Fyn	No	N.A.	[46]	0.48X Mean
GGEFpYGYMTMD	IRS-2 (Y540)	Plcγ_C	Yes	N.A.	[111]	Dapp1, Grb7
PNGDpYLNVSPS	IRS-2 (Y766)	Grb2	Yes	N.A.	[112]	Sh2d1b, Vav1
SNQEpYLDLSMP	FGFR1 (Y766)	Shb	No	N.A.	[113]	Similar peptide to Y760 of FGFR3 but has weak binding, 0.45X Mean
		Plcγ	No	N.A.	[55]	PLCγ_N – 0.52X Mean
						PLCγ_C – 0.21X Mean
THDLYMIMREA	FGFR3 (Y724)	Sh2b	No	N.A.	[114]	0.44X Mean
STDEpYLDLSAP	FGFR3 (Y760)	Sh2b	No	N.A.	[114]	0.46X Mean
VSEEYLDLRLT	FGFR4 (754)	Plcγ	No	N.A.	[115]	SHD
QVHTpYVNTTGV	FRS2 (Y196)	Grb2	Yes	N.A.	[116]	Abl2, Gads, Grap, PI3K1_C
NKLVpYENINGL	FRS2 (Y306)	Grb2	Yes	N.A.	[116]	Grap, Grb7, Sh2d2a
ALLNpYENLPSL	FRS2 (Y349)	Grb2	Yes	N.A.	[116]	Abl1, Abl2, Gads, Grap, Grb2, Grb7, PI3K1_C, Sh2d2a, Sh3bp2
PMHNpYVNTENV	FRS2 (Y392)	Grb2	Yes	N.A.	[116]	Gads, Grap
RQLNpYIQVDLE	FRS2 (Y436)	Ptpn11_N	Yes	N.A.	[117]	Itk, Mist, PLCγ_C, Rasa1_N, Shb, Shd, She, Syk_C
NPGFpYVEANPM	PLCγ1 (Y783)	Plcγ_C	No	N.A.	[118]	PLCγ_C at 1.03X mean; Zap70_N
EQDEYDIPRHL	p130Cas (Y234)	Crk	Yes	N.A.	[87]	Brk, Crkl, Fyn, Lck, Lyn, Shc1, Yes
PQDIYDVPPVR	P130Cas (Y249)	Crk	Yes	N.A.	[87]	Crkl, Zap70_N
WMEDpYDYVHLQ	p130Cas (Y664)	Nck1	Yes	N.A.	[87,119]	Bcar3, Brk, Crk, Crkl, Dapp1, Fer, Grb7, Matk, PI3K1_N, Rasa1_N, Rasa1_C, Sh3bp2
		Bmx	Yes	N.A.	[120]	Itk
		Src	No	K_D_ = 25-46nM	[119,121]	2.45X mean
		Lck	No	N.A.	[121]	2.03X mean
PPALpYAEPLDS	p62DOK1 (Y296)	Rasa1_N	Yes	N.A.	[122]	Abl1, Nck1, Vav1, Zap70_N
		Rasa1_C	Yes	N.A.	[122]	
QDSLpYSDPLDS	p62DOK1 (Y315)	Rasa1_C	Yes	N.A.	[122]	Abl1, Blk, Crk, Lck, Lyn, Nck1, Src, Zap70_N
EDPIpYDEPEGL	p62DOK1 (Y362)	Nck1	Yes	N.A.	[123]	Blk, Hck, Lck, Lyn, Shc1, Src
		Abl1	Yes	N.A.	[124]	
KEEGpYELPYNP	p62DOK1 (Y398)	Rasa1_C	Yes	N.A.	[123]	Abl1, Blk, Brk, Fgr, Fyn, Hck, Lck, Lyn, Nck1, Sh2d1b, Shc1, Ship2, Src, Vav1, Yes
		Rasa1_N	Yes	N.A.	[123]	

A set of control peptides with previously reported SH2 domain targets was included on each array. We further identified a set of literature-reported interactions between specific peptides present on the arrays and SH2 domains used in this study. Peptide sequence is indicated along with the source protein and corresponding position of the relevant phosphotyrosine residue. SH2 domains that were expected to bind to each peptide are noted along with the observed array-positive status. Measured equilibrium dissociation values (K_D_) or relative affinity (IC_50_) values reported in the literature are noted. Additional array-positive SH2 domains identified as interacting with each peptide are also indicated along with explanatory comments

N.A. – No Affinity Determined, # - IC50 Relative Affinities.

To assess the potential network of SH2 domain interactions we selected 50 SH2 domains representing 28 of the 38 families of SH2 domains (Figure 1B) all of which we have previously shown can be expressed and purified [23]. These include a number of extensively studied SH2 domains (Src, Grb2, PLCγ), as well as a number of less studied SH2 domains from proteins such as Shd, She, Shf, Slnk (Sh2d6), Sh2d1a (SAP), Sh2d1b (Eat-2), and Brdg1. To address potential variability in specificity within families we employed all members from the SHB, CRK, GRB2, SRC and ABL families (families are indicated with complete Capitalized lettering).

SH2 domains were arrayed as GST fusion proteins and detected using anti-GST primary antibodies and near-infrared labeled secondary antibodies. In an effort to present a dataset with minimal false positives, we chose an empirical cutoff based on the array average across all peptide spots to classify interactions (Figure 1A). In cases where the intensity of the signal for an individual SH2-domain binding event exceeded the mean intensity of all the peptides on the membrane by three-fold were scored as “array positives” [22]. Non-binding was judged in cases where the intensity of a spot was less than the mean intensity of all spots on the membrane and these were scored as “array negatives”. Peptides with signal intensities between 1X and 3X mean were scored as “indeterminate” and ascribed as neither array positive binding interactions nor array-negative non-binders. Analysis of the distribution of SH2 domain interactions per phosphopeptide revealed that our dataset possessed a bimodal distribution, with a significant number of peptides binding to many SH2 domains (Additional file 2: Figure S2). This signature may be indicative of promiscuity differences between phosphopeptides or there may be a subset of peptides which interact in a nonspecific fashion with either the GST fusion tag or one of the antibodies used for detection, resulting in false positives. Consistent with our goal of reducing the errors associated with identifying false-positives, we probed three separate arrays with three separate preps of the GST fusion tag alone. Potentially non-specificly interacting peptides (so-called ‘sticky’ peptides) were identified as any that bound to GST with above mean intensity in two out of three separate trials. This approach identifies any peptides which interact with GST or either of the recognition antibodies, a known confounding factor for downstream analysis [44]. This conservative approach allows us to score many significant peptides as ‘binders’ which may have been indeterminate before when incorporating the ‘sticky’ peptides into the array average. This resulting in discarding 40 peptides representing 382 potential interaction pairs as non-selective and resulted in a dataset of substantially higher quality.

### Validation by orthogonal assays and literature-verified interactions

To verify the binding results obtained from addressable peptide arrays we employed an orthogonal method of determining SH2 interactions with peptide ligands. We measured the dissociation constants of 60 binary SH2-peptide pairs in solution by fluorescence polarization (Table 2, Additional file 2: Figures S3A-C). In all cases array-positive interactions were of high affinity (range 0.18 μm – 5.8 μM, median K_D_ = 2 μM), while array negative interactions were demonstrably lower affinity (median K_D_ > 30 μM) (Figure 1C). This suggests a low false-positive rate and indicates that array-positive interactions correspond to high affinity binding events at a high frequency.

**Table 2:** Measured affinity values

**Peptide sequence**	**Protein/position**	**Expected partner**	**Array positive**	**Affinity (K**_**D**_**)/relative affinity (IC**_**50**_**)**	**References**	**Comments and other SH2 domains bound**
AEDVpYDVPPPA	p130Cas (Y362)	Abl2	No	K_D_ = 14 μM	This Study	
		Crk	Yes	K_D_ = 0.35 μM	This Study; [87]	Fyn, Hck, Src
		CrkL	Yes	K_D_ = 0.99 μM	This Study	
		Nck1	Yes	K_D_ = 0.93 μM	This Study	
		Ptpn11_N	No	K_D_ > 50 μM	This Study	
		Shc1	No	K_D_ = 32.8 μM	This Study	
		Ship2	No	K_D_ = 16.5 μM	This Study	Below Threshold (1.91X Mean)
SPGEpYVNIEFG	IRS-1 (Y896)	Abl1	No	K_D_ = 7.48 μM	This Study	Blk Dapp1 Fgr Grb7 Itk Mist Pi3k1_N Pi3k1_C Plcg1_C Ptpn11_N Rasa1_N Rasa1_C Sh2b Sh2d2a Shb Shf Shd She Syk_C Vav1 Yes
		Abl2	No	K_D_ = 3.66 μM	This Study	
		Crk	No	K_D_ > 20 μM	This Study	
		Grb2	Yes	K_D_ = 0.8 μM	This Study [92,108,109]	Gads Grap
				K_D_ = 35nM		
		PI3K1_N	Yes	K_D_ = 2.68 μM	This Study	
		Plcg2_C	Yes	K_D_ = 2.92 μM	This Study	
		Ptpn11_N	Yes	K_D_ = 2.08 μM	This Study	
				IC_50_ = 4.8 ± 1.0 μM	[107,108]	
		Sh2b	Yes	K_D_ = 3.58 μM	This Study	
		Src	No	K_D_ = 2.21 μM	This Study	
		Tenc1	No	K_D_ = 24 μM	This Study	
NGLNpYIDLDLV	IRS-1 (Y1179)	Abl1	No	K_D_ = 22.2 μM	This Study	Itk Plcg1_C Rasa1_N Sh2b Shb Shf Shd She Yes
		CrkL	No	K_D_ > 50 μM	This Study	
		Grb2	No	K_D_ > 50 μM	This Study	
		PI3K1_N	No	K_D_ = 15 μM	This Study	
		Ptpn11_N	Yes^3^	K_D_ = 3.0 ± 0.60nM	[95,108,110]	^3^Tandem SH2 domains of Ptpn11 was used to bind to the tandem motif of IRS-1
		Ptpn11_N	Yes	IC_50_ = 1.1 ± 0.5 μM	[107]	
		Src	No	K_D_ = 9.94 μM	This Study	
GVSEpYELPEDP	FGFR1 (Y463)	Brk	Yes	K_D_ = 0.38 μM	This Study	
		Crk	No	K_D_ = 44 μM	This Study	
		CrkL	No	K_D_ > 50 μM	This Study	
		Itk	Yes	K_D_ = 2.74 μM	This Study	
		Nck1	Yes	K_D_ = 2.45 μM	This Study	
		Rasa1_N	No	K_D_ = 1.54 μM	This Study	
		Rasa1_C	No	K_D_ > 17 μM	This Study	
		Src	No	K_D_ = 6.85 μM	This Study	
		Vav1	Yes	K_D_ = 1.87 μM	This Study	
STDEpYLDLSAP	FGFR3 (Y760)	Abl2	No	K_D_ = 27 μM	This Study	
		PI3K1_C	No	K_D_ = 2.14 μM	This Study	
		Plcg2_N	No	K_D_ > 50 μM	This Study	
		Plcg2_C	No	K_D_ = 7.49 μM	This Study	
		Ptpn11_N	No	K_D_ =25 μM	This Study	
		Sh2d1b	No	K_D_ = 5.3 μM	This Study	2.52X Mean, Lck
		Shb	No	K_D_ > 50 μM	This Study	Below Threshold, 0.23X mean
		Rasa1_C	No	K_D_ > 17 μM	This Study	
KEEGpYELPYNP	p62DOK1 (Y398)	Abl1	Yes	K_D_ = 5.77 μM	This Study	Blk Brk Fgr Fyn Hck Itk Lck Lyn Nck1 Pi3k1_N Sh2d1b Shc1 Ship2 Src Vav1 Yes
		Abl2	Yes	K_D_ = 3.47 μM	This Study	
		Brk	Yes	K_D_ = 0.42 μM	This Study	
		Crk	No	K_D_ = 23.7 μM	This Study	
		Fer	No	K_D_ = 13.3 μM	This Study	
		Fgr	Yes	K_D_ = 2.04 μM	This Study	
		Itk	Yes	K_D_ = 5.43 μM	This Study	
		Nck1	Yes	K_D_ = 4.37 μM	This Study	
		PI3K1_N	Yes	K_D_ = 1.85 μM	This Study	
		PI3K1_C	No	K_D_ = 13 μM	This Study	
		Ptpn11_N	No	K_D_ > 50 μM	This Study	
		Rasa1_N	Yes	K_D_ = 0.41 μM	This Study	
				N.A.	[123]	
		Rasa1_C	Yes	K_D_ = 1.39 μM	This Study	
				N.A.	[123]	
		Sh3bp2	No	K_D_ = 1.68 μM	This Study	
		Shc1	Yes	K_D_ = 5.74 μM	This Study	
		Src	Yes	K_D_ = 5.06 μM	This Study	
		Vav1	Yes	K_D_ = 4.73 μM	This Study	

Select SH2-peptide interaction pairs were confirmed by fluorescence polarization solution-binding. Additional affinity values from published sources have been included and listed accordingly.

**Figure 2:**
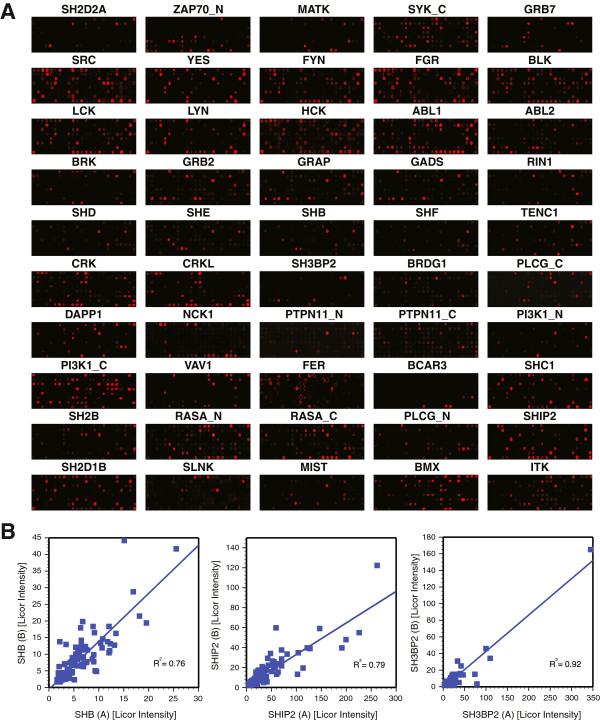
**Addressable peptide arrays reveal SH2 domain selectivity. **(**A**) 50 SPOT arrays panned against 50 GST-SH2 domains reveals the highly selective nature of SH2 domain phosphopeptide interactions. Interactions were detected using anti-GST antisera and Alexa Fluor-680-labeled anti-goat secondary antibody and the intensity of signals recorded using LiCor Odyssey. (**B**) Two separate peptide arrays were probed with independent SH2 domain preparations for three SH2 domains (SHB, SHIP2, SH3BP2). The scatter plot reveal some variability between the independent SPOT experiments yet revealing a strong correlation coefficient (R^2^).

Probing of arrays individually with each of 50 SH2 domains provides a snapshot of SH2 specificity (Figure 2A). As we have previously shown, this method is highly reproducible [22]. Independent peptide arrays and protein preparations reveal high reproducibility for the select SH2 domains (Shb, Ship2, Sh3bp2) (Figure 2B). To confirm interactions between full-length proteins we performed a set of GST-SH2 pull-down experiments of CHO stably expressing InsR and IRS-1 with or without stimulation with insulin (Additional file 2: Figure S4). These lysates were incubated with GST-SH2 domains and precipitated using glutathione-agarose beads to identify SH2 domains that were capable of precipitating phospho-IRS1 or phospho-InsR. This confirmed previously described interactions such as those involving the PI3K_C, Shp2_N and Fyn (as well as related Src and Itk) SH2 domains [45-47]. In addition, interactions observed on the peptide arrays were confirmed for Rasa1, Vav1, and Abl2 and PLC-γ1.

The literature is a rich source of detailed interactions that provide potential validation. Since the discovery of the SH2 domain in 1986 [48], detailed study has uncovered a large set of SH2 interactions. Any high-throughput technique would expect to capture most of these interactions, and failure to do so may be taken as evidence of false-negative results. Each of our addressable peptide arrays included a set of 12 designed control peptides for which 22 reported interactions covered a range of K_D_ values. In addition, we noted 43 interactions with the 13 signaling proteins represented on the arrays reported in UniHI [49] from the interaction databases of MINT [50], BIND [51], HPRD [52], and DIP [53]. Of the 22 designated control interactions, 18 were noted as array-positive (Table 1). Of the remaining four expected interactions, three have measured affinities, and in all cases the equilibrium dissociation constant is weaker than 16 μM. All of the array-positive interactions for which affinity is reported have K_D_ values stronger than 4.1 μM. Thus, this control set suggests an approximate threshold of binding in the range of 10 μM ± 5 μM. Of the 43 database-reported interactions, most were array positive and of those that were not array-positive, a number were just sub-threshold and judged to be indeterminate (Table 1). The ability to recapitulate the vast majority of known (literature-reported) interactions and to verify novel interactions by orthologous methods is indicative of a high quality dataset [54].

### Reconciling conflicts with other datasets

As noted above, this study performs well in terms of reproducing the literature reported interactions between the 50 SH2 domains tested and the 13 proteins represented on the addressable arrays (Table 1). A handful of differences with literature-reported interactions must, by necessity, be reconciled. Our assumption is that a high-throughput (HTP) study such as this one should capture upwards of 85% of known (literature reported) interactions and that results that differ from low throughput studies described in the literature should be subject to further testing to identify the nature of the discrepancy and reveal any weakness in the HTP dataset [55]. We examined a set of potential discrepancies and found that in each case our dataset held up well. For instance, FGFR1 Y-766 (SNQEpYLDLSMP) is reported to bind to PLCγ1 in a pTyr dependant manner based on mutational analysis of FGFR1 [55,56]. We tested the PLCγ2 SH2 domain with an analogous peptide from FGFR3 Y-760 (STDEpYLDLSAP) and failed to detect any interaction. Direct measurement of peptide binding to either the PLCγ2_N or PLCγ2_C SH2 domain by fluorescence polarization in solution also failed to detect an interaction, supporting the results on the array (Table 2, Additional file 2: Figure S3). This may imply that either this is a binding event specific to PLCγ1 (and not PLCγ2), or that the interaction reported at the level of the full-length protein may be more complex, perhaps requiring secondary contact sites that are not available within the context of the short peptide used in the current study. In several other cases, literature-reported interactions that were array-negative turned out to be interactions with IC50 or K_D_ values above 10 μM (Table 1). It is likely that a few low micromolar or even sub-micromolar binding events could be assigned as array-negative in our study due to synthesis yield heterogeneity and the fact that we are limited to arraying at one concentration (0.25 μM in this study). We decided to design an empirical reporting scheme that was conservative, sacrificing many true positives in order to limit false positives, which would have naturally arisen in the process of trying to minimize false negatives. We have made an effort to limit false negatives to those of lower affinity, and we are aware of no instance in our dataset of a sub-micromolar affinity interaction being scored as array-negative.

Many high-affinity interactions, such as the interactions between the Src and Lck SH2 domains and p130Cas pY-664, fell into our array-indeterminate set (1x-3x mean), likely due to the synthesis efficiency and accessibility of these particular peptides and the semi-quantitative nature of the system. Indeed, many of the peptide-SH2 interactions that fall in the indeterminate set are likely to be real binders. Some surprising differences between SH2 domains can be reconciled this way. For instance, comparing between the Abl1 and Abl2 SH2 domains there is a significant difference in array positive interactions between the two. This is surprising considering the sequence similarity between the two domains. Because of the heterogeneities inherent in this study design as indicated above and the similarities between the two proteins, discrepancies of this sort likely represent false negatives. In total, the limited number of incongruities between the current data set and the literature are thus largely reconcilable.

A high-throughput binding study reported interactions between a large set of SH2 domains and phosphopeptides within four receptor tyrosine kinases (including IGF-1R and FGFR1) overlaps with the present study [57]. Our dataset only validates 5 of 51 of these interactions and describes 6 additional interactions not reported in that study. This disagreement is in contrast to the high degree of consensus between the present study and a wide range of previous studies (Table 1). We examined a number of the interactions reported by Kaushansky A et al. using a combination of an orthologous experimental approach, comparison to consensus binding motifs, and literature validation. As noted above, SH2 domains have well described binding motifs and adhere to these remarkably well in the current study. Kaushansky A et al. report a large number of interactions that do not approximate the binding motifs to which the corresponding SH2 domains are known to be capable of binding. In addition, SH2 domains make use of contextual sequence information and non-permissive residues that block binding in order to improve selectivity [22]. For example, the Grb2 family has a very strong preference for an asparagine residue at the +2 position and will not tolerate a proline residue at the +3 position [19-22]. Kaushansky A et al. report a series of Grb2 interactions with peptides that do not contain the required permissive residues, and furthermore many that contain strong non-permissive residues (Additional file 2: Figure S7 and Table S3). Similarly, Crk SH2 requires a +3 Leu or Pro yet this motif is absent in many of the Crk SH2 binding peptides reported by Kaushansky et al. Indeed, the 46 interactions reported by Kaushansky et al. that we fail to confirm overwhelmingly contain peptides that lack conformity to the consensus motifs to which the cognate SH2 domains are known to interact [19,20,29]. In addition, a number of apparent “hub” peptides reported in Kaushanky et al. contain cysteine residues (eg. FGFR1 pY-583, FGFR1 pY-605, FGFR1 pY-730), and the interactions were probed in the absence of reducing agents [57,58]. In the present study, binding was assayed in the presence of 1 mM DTT and peptides containing cysteine residues were substituted with serine [59]. Kaushansky et al. provide no corroboration of their results by either orthogonal assay or literature validation, while the present study provides extensive corroboration.

Even in the cases where our data overlap, the reported apparent K_D_ values reported by Kaushansky et al. appear inconsistent with direct measurements conducted using well controlled solution binding measured by fluorescence polarization [57]. For example, Kaushanskyet al. report a K_D_ of 175nM for the interaction between Rasa1-N-SH2 and FGFR1 pY-463 while we measured a K_D_ of 1.54 μM by fluorescence polarization (Additional file 2: Figure S7), Additionally, there are 6 interactions that we report that are not noted by Kaushansky et al. We picked one of these binary pairs at random, the interaction between Crk SH2 and FGFR1 pY-463, and tested binding in solution. We measured K_D_ of 380 nM for this interaction, validating this binding event.

Taken as a whole, comparisons with the literature validate the results presented in this study. Non-array-positive literature-reported interactions tend to fall into three categories: 1) low affinity interactions; 2) near misses that are array-indeterminate and thus just below threshold; or 3) cases where orthogonal measurement confirms no interaction at the level of the individual SH2 domain and 11-mer phosphopeptide. Comparison with an SH2 domain array study reveal limitations in that technique and suggest that SH2 domain arrays on glass substrates may suffer from a high rate of false positive and false negative interactions. This is consistent with results from the same group investigating PDZ domain binding using a similar protein microarray method which concluded that the technique resulted in a false positive rate of approximately 50%, and poor correspondence between array-estimated and solution-binding measured equilibrium-dissociation values [60-62].

### Metadata-rich interaction maps

**Figure 3:**
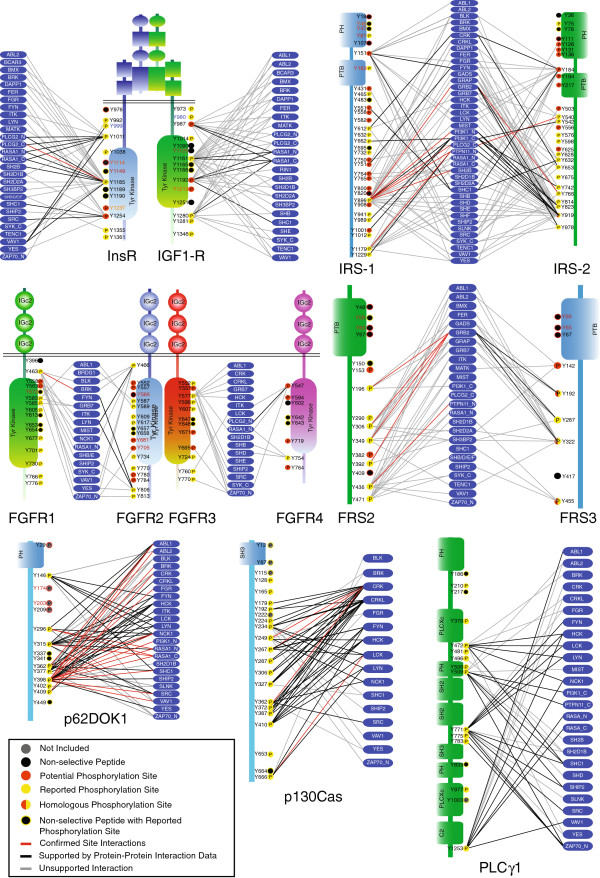
**High-resolution interaction maps detail an SH2 domains potential interactome.** A phosphotyrosine interactome for 13 proteins involved in FGF-family and Insulin-family signaling and 50 SH2 domain partners. Phosphotyrosine peptides are indicated by their position within their host protein and color-coded as either PhosphoSite reported phosphorylation sites (yellow); sites not reported as phosphorylated (red); sites not reported to be phosphorylated but where a closely related site on a paralogous protein is known to be phosphorylated (red/yellow); or the peptide was discarded as non-specific (black). Interactions between the vertices of SH2 domains and phosphopeptides identified in this study are indicated as edges (lines) and color-coded according to the level of support provided by previous studies: if the precise phosphorylation site has been reported to interact with the noted SH2 domain the edge is denoted in red. A black line is representative of proteins that are reported to interact defined by interaction databases including HPRD, BIND, MINT and DIP, but the site of interaction is unknown. SH2 interactions not confirmed by literature but whose binding is greater than 3X mean on the array are represented with grey lines.

Probing arrays with 50 SH2 domains identifies a total of 529 array-positive interactions, together with 5949 array-negative and 1122 indeterminate SH2-ligand pairs. Array-positive interactions between SH2 domains and pTyr sites map the potential SH2 interactome. The connections between SH2 domains and InsR, IGF-1R, IRS-1, IRS-2, FGFR1, FGFR2, FGFR3, FGFR4, FRS2 and FRS3 together with p130Cas, PLCγ1 and p62DOK1 highlight a wide range of putative SH2 interactions within the immediate FGF and Ins/IGF-1 signaling networks (Figure 3). The prediction of novel interactions comes with the inherent caveat that a given SH2 protein would need to be co-expressed with its interaction partner. For example, Grap and Gads are expressed only in certain hematopoietic cells [63,64]. Interactions recorded for the SH2 domains of Gads and Grap are not useful for predicting interactions in other cell types but may be considered as supporting data for the interactions of the closely related Grb2 SH2 domain. The similar specificity of the SH2 domains of Grb2, Gads and Grap results in an overlapping set of target peptides where the independent binding of all three SH2 domains increases our confidence that this peptide is in fact a high-quality ligand for this class of SH2 domains.

To enhance the interaction maps derived the current study, we incorporated multiple layers of additional data gleaned from a variety of sources. Specific phosphopeptides reported in the PhosphoSite database are noted for each of the 13 target proteins in Figure 3 (Additional file 1: Table S1) [65]. Reported phosphorylation remains a moving target, particularly as certain sites may be phosphorylated only in certain tissues or transiently upon recruitment of specific kinases [33]. In cases where phosphorylation of a tyrosine residue has been reported, we assume that region to be solvent accessible and capable of interactions. If phosphorylation has not been reported solvent accessibility may be considered as a minimal threshold for phosphorylation and SH2 domain binding. This is with the caveat that certain residues, such as the activation loop tyrosine in the kinase domain of the InsR and IGF-1R are buried in the inactive state but become phosphorylated and solvent exposed in the activated state. The phosphorylated and exposed activation loop is then able to bind to SH2 domains [66]. Given the dynamic nature of protein structures and the ability of buried residues to become exposed upon structural rearrangement, one cannot presuppose that buried residues never become exposed. Nonetheless, solvent accessibility provides an additional level of support for potential phospho-dependent interactions in cases where phosphorylation has not been reported. Existing structures provide a greater level of confidence in such interactions while at the same time identifying potential anomalous interactions with buried peptides. The Gerstein Accessible Surface algorithm was employed to calculate the accessible molecular surface [67,68] of each tyrosine residue within structure files PDBID:1IRK, 2DTG, 1P4O, 1K3A, 1IRS, 1QQG, 2FGI, 2PVF, 2PSQ, 1XRO, 2YS5, 2YT2, 2 V76, 1WYX, 1HSQ, and 2HSP that represent regions of InsR, IGF-1R, IRS-1, FGFR1, FGFR2, FRS2, FRS3, p62DOK, p130Cas and PLCg in various conformations (Additional file 2: Table S4). Sites that fell below the threshold of the minimally accessible phosphorylation site (excluding the activation loop tyrosine) are marked in orange text for the residue number in Figure 3. Many of these sites are also excluded as non-specific interaction sites, likely reflecting their hydrophobic nature. Inclusion of structural data, where available, makes use of a significant resource to interpret potential pTyr interaction data.

Previously reported specific SH2-phosphopeptide interactions confirmed in this study (Table 1) are highlighted as red lines (Figure 3) and represent the highest confidence interactions. Noted as black lines are cases for which protein-protein interactions have been reported in MINT [50], BIND [51], HPRD [52], and DIP [53], without reference to specific binding sites or direct involvement of an SH2 domain. Interactions noted in the current study that are not listed in any of the major interaction databases, are represented as grey lines.

### Position weighted matrices define physiological ligand specificity

**Figure 4:**
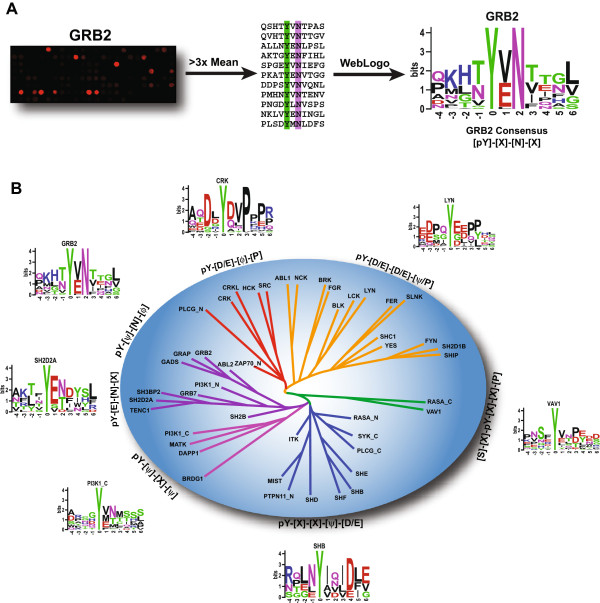
**Specificity for physiological peptides defines functional groups of SH2 domains. **(**A**) Grb2 SH2 domain positive peptides are highlighted and then represented as an EDSM logo. See Figure S5 for EDSM logos of all tested SH2 domains. (**B**) An unrooted dendrogram clusters families of SH2 domains related by similar binding patterns. A distance matrix between EDSMs was computed and used to generate an unrooted distance tree (see Figure S6). This is artistically represented as a dendrogram with general specificity information overlaid and functional classes denoted by branch color.

To represent the specificity of SH2 domains in this study we define position weighted matrices (PWMs) based on the array-positive peptides. PWMs such as the position-specific scoring matrix (PSSM) [21] are a well-established method to describe biding motifs. In a PWM, each matrix column describes the probability that a given amino acid will be found at that ligand position. The PWM may also be visualized as a sequence logo [69] (Figure 4A). The 192 physiological peptides represented on the arrays in this study do not conform to a random distribution of residues at each position. To compensate for this the matrices were corrected for the prevalence of amino acids residues at each position in the total data set. In addition, the absence of binding to a given peptide may provide data on inhibitory effects of specific residues. For instance, lack of binding may result from either the absence of critical permissive residues or from the presence of inhibitory residues at specific positions [22]. To make use of both array-positive and array-negative data we corrected for frequency of occurrence of a given residue at each position using the array-positive peptides (posPWM). This is compared to a PWM of the expected frequency of all peptides, excluding non-specific peptides (exPWM). The scoring matrix that results from subtracting exPSSM from posPSSM expresses the deviation observed in the array-positive data from that of all specific peptides on the array. We term this the expectation-deviation scoring matrix (EDSM).

(1) $$\left[\text{EDSM}\right]=\left[\text{posPWM}\right]–\left[\text{exPWM}\right]$$

By expressing differences between peptides that bind specifically and the peptide set as a whole, the EDSM attempts to compensate for any inherent bias arising in the relatively small set of non-random peptides drawn from physiological proteins. The EDSM for each SH2 in this study is visualized using sequence logos (Additional file 2: Figure S5) and condensed into a generalized statement of physiological specificity in the form of a regular expression (Table 3). A distance matrix comparing the EDSMs for the physiological specificity of the SH2 domains describe families of SH2 domains related by their preference for physiological ligands (Additional file 2: Figure S6). This is represented as an unrooted tree of SH2 domain specificity (Figure 4B). Six classes of general specificities are displayed among the SH2 domains tested in this study revealing similarity among SH2 domains within the same family (eg Grb2, Gads, Grap) and across different families (Sh2d1b, Ship2) but also subtle differences (eg Abl1 and Abl2). Although the EDSM is informed by both permissive and non-permissive effects, the limited dataset afforded by the addressable arrays in this study limits the utility of the resulting matrices for extrapolating information on non-permissive residues.

**Table 3:** Specificities obtained using Physiological Ligands

**SH2 Domain**	**Specificity**
ABL1	[pY] [D/E/S] [D/E] [P/N/D/E]
ABL2	[pY] [V] [N/Q]
BLK	[pY] [D/E/ϕ] [D/E/L] [P/I]
BRK	[pY] [D/E] [X] [D/E/ϕ]
CRK	[D] [X] [pY] [D] [V/L] [P] [P]
CRKL	[D] [X] [pY] [D] [ϕ] [P] [P/R]
DAPP1	[pY] [X] [X] [D/E/ϕ] [E]
FER	[D/E] [D/E] [pY] [D/G] [D/E] [ϕ]
FGR	[E] [P/D/E] [X] [pY] [D/E/G] [X] [D/E/ϕ] [Y]
FYN	[pY] [X] [D/ϕ] [ϕ]
GADS	[pY] [V] [N]
GRAP	[pY] [V/E] [N]
GRB2	[pY] [V/E] [N]
GRB7	[pY] [E] [N/Y]
HCK	[D/E] [D/P] [X] [pY] [D/E/G] [D/E/ϕ] [P/I/L]
ITK	[pY] [ϕ] [X] [D/ϕ]
LCK	[pY] [D/E/G] [D/E] [P/L] [P]
LYN	[pY] [D/E/G] [D/E] [P] [P]
MIST	[pY] [ϕ] [ζ] [ϕ] [D/E] [ϕ]
NCK1	[pY] [D/E] [E/L] [P/V]
PI3K1_N	[pY] [V/D] [X] [I/M/V]
PI3K1_C	[pY] [V/M/E] [N/T/M] [M]
PLCG1_C	[pY] [ϕ] [X] [D/E]
PTPN11_N	[pY] [ϕ] [X] [ϕ] [D/E] [ϕ]
RASA1_N	[pY] [ϕ] [X] [D/ϕ]
RASA1_C	[pY] [X] [X] [D/E/ϕ]
SH2B	[pY] [X] [X] [D/E/ϕ]
SH2D1B	[pY] [X] [X] [ϕ]
SH2D2A	[pY] [E] [N/T] [D/ϕ]
SH3BP2	[pY] [D/E] [N] [V]
SHB	[pY] [ϕ] [X] [ϕ] [D/E] [ϕ]
SHD	[pY] [ϕ] [X] [ϕ] [D/E] [ϕ]
SHE	[pY] [ϕ] [X] [ϕ] [D/E] [ϕ]
SHF	[pY] [ϕ] [X] [ϕ] [D/E] [ϕ]
SHC1	[pY] [D/E/G] [D/E/ϕ] [ϕ]
SLNK	[pY] [G/D/V] [D/T] [D/ϕ]
SRC	[D/E] [X] [X] [pY] [D] [D/E/ϕ] [P/I]
SYK_C	[ϕ] [pY] [V] [X] [D/E/ϕ] [D/E]
TENC1	[pY] [E]
VAV1	[pY] [V/E/L] [X] [P]
YES	[pY] [D/E/G] [D/E/ϕ] [ϕ]
ZAP70_N	[P] [X] [pY] [X] [X] [ψ/ϕ]

The general specificity information is obtained from the arrays is expressed in a regular expression form. Amino acid residues are indicated by their single-letter codes. Groups of amino acids are noted as φ = hydrophobic residues (Val, Ile, Leu, Phe, Trp, Tyr, Met); ψ = aliphatic residues (Val, Ile, Leu, Met); ζ = polar residues (Asn, Gln, Ser, Thr, Glu, Asp, Lys, Arg, His).

## Discussion

**Figure 5:**
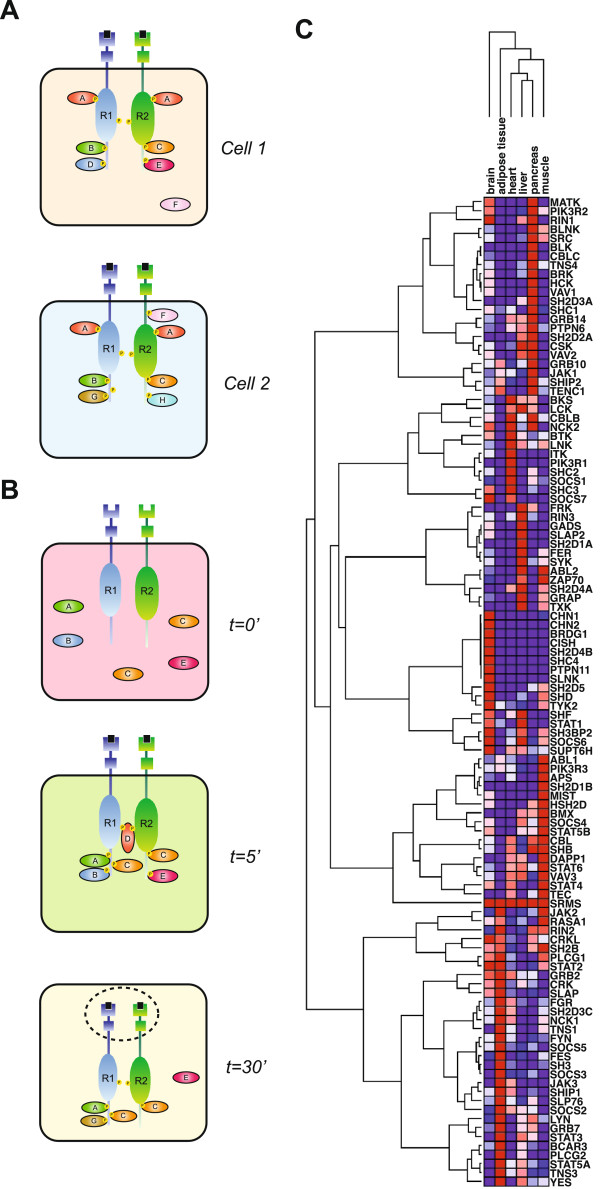
**Tissue co-expression and microstate of the Insulin/IGF-1 system.** Protein interaction microstates across different cell types and across time and space. (**A**) Co-expression between receptors and SH2 domains can influence the microstate of a specific tissue. (**B**) Phosphorylation of receptors under stimulation conditions can determine the temporal and spatial events of SH2 ligand binding within a cell. (**C**) Hierarchical clustering of the insulin responsive tissue expression levels for human SH2 domain-containing genes.

The analysis of SH2-mediated interactions with peptide ligands representing the receptors and substrate proteins of the insulin, IGF-1 and FGF systems described herein, reconstructs the set of potential phosphotyrosine-mediated interactions that determine the capacity of these systems to recruit signaling proteins upon activation. The potential interactome outlines the possible signaling states that may participate in signaling. Among the factors that determine the possible signaling networks initiated by activated receptors are 1) the available set of SH2 proteins expressed in specific cells; and 2) the capacity of phosphorylated receptor and scaffold sites to recruit those SH2 proteins. The 111 SH2 domain proteins extant in the human genome vary extensively in their tissue and cell specific expression [15,36]. In some cases these expression differences are drastic and even define highly tissue-specific signaling networks such as those in B- and T-lymphocytes [14,15]. Among the 38 SH2 families, 33 possess at least one gene duplicate allowing a duplicate copy to acquire new functions such as specialized tissue functions or novel scaffolding capabilities [70]. The expression of a family member in one tissue may perform a redundant function to its paralog in another tissue but may also diverge in terms of functions (Additional file 2: Table S5). The potential interactome for SH2 domains indicates many cases of potential overlap in binding, resulting in pTyr sites that may act as hubs for multiple interactions or serve distinct binding functions in cases where the SH2 complement varies in different cells. The varied potential interaction permutations, or microstates, in turn, are the basis for highly cell-specific signaling outcomes from discrete signal inputs [34]. In simple terms, differences in the available phosphorylated tyrosine sites as well as in the expression of SH2 domain proteins themselves has the potential to furnish related but distinct signaling events in responses to the same input signal (Figure 5A). Currently the phosphorylation dataset available from PhosphoSite and PhosphoELM provide only a static view of receptor and scaffold phosphorylation. Even within a cell, the available complement of pTyr sites and locally available SH2 domain proteins may vary over the lifetime of a signal. Protein interaction microstates may differ according to the intensity of ligand stimulation and change as signaling complexes move within the cell, for instance as receptors are internalized on signaling endosomes (Figure 5B). For example, Grb10 and Grb14 are closely regulated adaptor proteins that share similar functions by binding to InsR and negatively regulating insulin signaling. While both genes share high expression in the pancreas, expression varies among adipose, liver and the heart (Figure 5C). However, little is known about the temporal and spatial dynamics between these two adaptors. Recently studies utilizing multiple reaction monitoring (MRM) mass-spectrometry has been applied to the Grb2 adaptors to map the dynamic interaction states upon various growth factor stimulation [71]. Analyses of this type will allow us to better dissect the vast number of microstates among different tissues. Thus, potential interactomes represent crucial datasets to interpret cell and tissue specific signaling events. This is particularly relevant in human development and diseases such as cancer in which receptor tyrosine kinases are commonly over-expressed, sometimes by several orders of magnitude. In such pathologies, the primary signaling pathways may be titrated out and novel, normally non-physiological pathways may become activated. For instance, IGF-1R is either overexpressed or hyperphosphorylated and deregulated in a range of cancers and is currently one of the most studied molecular targets in the field of oncology yet direct targeting of IGF-1R has proven problematic due to it’s wide range of important physiological functions [72-74]. Under conditions of hyperphysiological abundance of IGF-1R pTyr sites available for SH2 binding, the potential interactome suggests the potential for non-canonical pathways to become activated, perhaps hinting at novel targets for therapeutic intervention.

Even in normal physiological circumstances of healthy tissues, the potential interactome may inform our understanding of tissue-specific signaling events. A variety of tissues can respond to insulin stimulation, including adipose, muscle, pancreas, liver, brain etc. [75,76]. SH2 domain-containing proteins vary widely in their expression in various cells and tissues (Figure 5C). While this likely represents only a piece of a much larger puzzle, it is conceivable that some of the observed tissue-specific responses and downstream signaling differences may relate to the available complement of SH2-containing signaling proteins and their ability to interact with available pTyr sites. In this way, the potential interactome and cell-specific expression combine to determine effective signaling networks.

### Consensus motifs and co-evolution

The interaction data also reveals the specificity of 50 SH2 domains for a set of physiological peptides. Typical binding motifs for SH2 domains describe the residues at positions +1 to +4 C-terminal of the essential phosphotyrosine [77-79]. SH2 domain peptide binding motifs have been described for a wide range of SH2 domains using peptide library approaches [19,20,29]. Binding motifs obtained from peptide library approaches represent optimal solutions unconstained by physiological parameters such as the confounding effects of kinases recognition or structural influences of native proteins. The motifs described herein represent binding to ‘real-world’ peptides and thus stand as a relevant contrast to peptide-library based data. However it should be noted that this dataset corresponds to a potential physiological interactome. Because all of the peptides haven’t been confirmed to be phosphyorylated *in vivo*, our interaction maps are best used in conjunction with the expanding mass spectrometry literature and their associated databases.

Broadly speaking, the SH2 consensus binding motifs identified from interactions observed using addressable arrays of physiological peptides are remarkably similar to the motifs described using peptide library approaches (Table 3). Yet binding specificities observed for physiological phosphotyrosine peptide ligands may in some cases represent more than the specificity of the isolated SH2 domain. The EDSM position weighted matrices noted in Additional file 2: Figure S5 reveal a number of cases in which the residues outside of the conventional window of residues at positions +1 to +4 appear to influence binding. Longer contact regions have been noted for certain SH2 domains in the past, though these are generally exceptions to the rule. For instance, the SH2 domain of SH2D1A/SAP binds to an extended peptide in the SLAM receptor comprised of residues −2 to +3 and shows a diminished dependence on phosphorylation of the tyrosine for binding [43]. Physiological peptide ligands co-evolve to allow recognition by their cognate SH2 domain partner, while also acting as competent substrates for their cognate kinases. In some cases, the observed specificity for physiological peptide ligands may therefore represent an amalgam of SH2 specificity, kinase recognition, and other factors. This may, for example, explain the apparent observed preference of the Crk SH2 domain for an Asp residue at the −2 position. The presence of an aspartic acid residue at the −2 position does not appear to contribute to Crk SH2 domain binding (Figure 4B), however, this may instead reveal a signature for a distinct event such as kinase recognition for a specific subset of physiological peptides. Indeed, a large number of tyrosine kinases have reported preference for acidic residues preceding the target tyrosine residue [80,81]. Not surprisingly, acidic residues are commonly observed in the EDSM logos for the SH2 domains (Additional file 2: Figure S5). In addition to acting as kinase substrates and SH2 domain binding sites, the peptide motif must also presumably be surface exposed, and potentially disordered prior to binding, and these factors may also contribute to the overall physiological peptide motif. Combining multiple motifs in computational searches has been shown to markedly increase predictive accuracy [82], suggesting that the inclusion of indirect components such as kinase specificity may make for a more robust predictor of SH2 interactions. While the current data set is relatively small in size, larger sets of data identifying physiological peptide interactions may provide useful data for investigating the overlapping influences of multiple events required for functional signaling based on overlapping motifs.

In our analysis we find that peptides reported to be phosphorylated in PhosphoSite are significantly more likely to have one or more SH2 domain-binding partners than peptide nodes that are not currently known to be phosphorylated. This is not surprising given that evolutionary pressure may be exerted to conserve critical binding sites. Conversely, given the specificity of SH2 domains, the chances of an SH2-interacting peptide occurring by chance within a non-phosphorylated peptide may be assumed to be relatively low. The more residues that must be specified to stipulate binding, the lower the probability is that this will occur spontaneously within a non-phosphorylated sequence. If only one key residue supported by one of two secondary residues was capable of allowing an SH2 domain to bind, then the chances of randomly generating an SH2 binding site centered around a given tyrosine residue are less than one in a hundred. Given the specificity observed for SH2 domains in this study, the likelihood of a random sequence encoding an SH2 domain ligand appears rather limited. The appearance of a small number of highly connected peptide nodes on sites not currently known to be phosphorylated raises the question of whether SH2 domain-binding might serve as means of predicting phosphorylation. Perhaps highly connected peptide hubs such as IRS1 Y-151, IRS2 Y-184, FRS3 Y-287 and FRS3 Y-322 predict phosphorylation. ScanSite predicts the first three of these sites as kinase substrates, while the sequence surrounding FRS3 Y-322 is identical to a known phosphorylation site on FRS2, suggesting that these may indeed turn out to be phosphorylated under appropriate conditions.

A high degree of selectivity for physiological ligands may itself be an outcome of evolutionary pressures, as has been noted for yeast SH3 domains. The Sho1 SH3 domain recognizes a binding peptide in Pbs1, and no other SH3 domain in the yeast genome cross-reacts with the Pbs1 peptide. SH3 domains from other species that have not been under evolutionary pressure to ignore this site exhibit less selectivity for the Pbs1 peptide [83]. A high degree of specificity among human SH2 domains, combined with cell-specific expression is consistent with the notion that evolutionary pressures drive selectivity of protein-ligand interactions.

### Comparison to the literature

In the quarter century since the SH2 domain was first described [48,84], hundreds of interactions have been described between SH2 domains and phosphotyrosine peptides. In many cases these have been subject to intensive biophysical analysis yielding a considerable set of bonafide interactions against which HTP studies can be validated. Placing new studies within the context of the extant literature is particularly important for systems levels studies for which validation is inherently limited. In the case of the 50 SH2 domains and 192 peptides included in this study, we confirmed 60 interactions by the orthologous method of fluorescence polarization. We compared our results to those reported in previous studies. In the case of carefully controlled studies that examine SH2 interactions, our results closely match the reported interactions (Table 1). However, our results did not match well against one large-scale interaction study conducted using SH2 domain arrays (Additional file 2: Table S3) [57]. Our results suggest that the SH2 protein micro-array results may suffer from high false-positive and false-negative rates and that the reported K_D_ values are likely inaccurate. This is consistent with other studies suggesting that protein microarray data is semi-quantitative and subject to false-positive results [60], particularly in the absence of orthologous validation

Several lessons may be taken from such results and suggest a set of standards that could be universally applied in future high throughput studies of protein-peptide interactions and these are explored in detail elsewhere [54]. First, proteins are fundamentally problematic in that they may easily lose binding activity. A set of positive controls is thus essential and should be present in every assay. Only about half of the SH2 domains express well as fusion proteins from bacteria [23]. The rest suffer from poor expression and lack reproducible binding activity, suggesting that any use of these SH2 domains in high-throughput *in vitro* binding studies may yield erroneous results. The present study used only 50 SH2 domains that have previously been shown to express well and exhibit good solubility and reproducible binding. A second issue relates to validation by orthologous method, to which the current study examines 60 binary pairs by the orthogonal method of solution phase fluorescence polarization binding, as well as a smaller set by GST-pulldown. A third consideration is agreement between HTP datasets and existing literature. Well-controlled studies reporting peptide-binding motifs for SH2 domains provide a wealth of data. SH2 domains bind to relatively specific motifs [19,29], and these provide excellent validation tools. Apparent interactions that do not match the known binding motifs are a cause for concern and should be further validated. As noted in Table 1, the dataset described in this study is in strong agreement with literature-reported interactions, and the variations can largely be rationalized.

### Concluding remarks

In examining SH2 domain interactions, we followed a systematic approach for systems-level interactome studies using orthologous validation and literature curation as a means of enhancing confidence in the experimental dataset. This results in a large set of high-confidence interactions that outline the potential interactome between 50 SH2 domains and 192 phosphopeptide sequences covering 13 proteins involved in FGF, Insulin, and IGF-1 signaling. The development of a detailed potential interactome for this set of signaling components represents an early step towards a more detailed understanding of cell-specific signaling networks. This stands to deepen our understanding of tissue-specific and disease-specific signaling networks that are predicated upon the varying and inevitably complex interpretation of the potential interactome by the available expressed interaction partners.

### Experimental procedures

#### Plasmids and recombinant proteins

A comprehensive list of 121 SH2 domains contained in 111 human proteins [14] served as the starting point for the assembly of a large set of SH2 domain clones. The cDNA clones for SH2 domains were obtained from ATCC except for those noted otherwise. A complete list of source DNA and SH2 clones is shown in Additional file 3: Table S2. SH2 domains were cloned into pGEX-2TK (Amersham Pharmacia) and verified by DNA sequencing. GST-fusions of SH2 domains were expressed in *E. coli* strain BL21 (Stratagene) at 37°C overnight and induced with 1 mM IPTG for 3 hours. Cells were centrifuged, resuspended in PBS and lysed by sonication. The cellular fractions were incubated with glutathione sepharose (Thermo Scientific) and washed with PLC lysis buffer (50 mM Hepes pH 7.5, 150 mM NaCl, 10% glycerol, 1% Triton X-100). SH2 proteins were eluted using 10 mM glutathione, 50 mM Tris HCl pH 8.0 and purified using the NAP-10 (Amersham Pharmacia) column system.

#### Peptide arrays

The peptide libraries were synthesized onto an acid hardened amino-PEG500 cellulose membrane #UC540 (Intavis, Germany) using an Intavis Multipep as described [41]. The estimated yield of peptide at each position was approximately 5 nmols. Addressable peptide arrays representing physiological peptides were composed of 192 peptides, each composed of 11 amino acid residues, corresponding to tyrosine-containing peptides from InsR, IGF-1R, IRS-1, IRS-2, FGFR1, FGFR2, FGFR3, FRS-2, FRS-3, PLCγ1, p130Cas, p62DOK1. Phosphotyrosine residues were located at the fifth position in singly phosphorylated peptides. In most cases Cys residues were replaced with Ser. The membranes were stored at −20 until use. The membranes were deprotected according to manufacturer instructions, using a 95% TFA, 3% TIPS, 2% H_2_O cocktail for three hours. Phosphotyrosine incorporation was assessed by incubation with anti-phosphotyrosine antisera 4 G10 (Upstate) and pY20 (Santa Cruz). Additional file 1: Table S1 indicates the array position, peptide sequence, protein source position, and comments on related peptides and synthesis problems.

#### SPOTs Analysis of SH2 domain specificities

All steps were carried out at room temperature unless otherwise specified. The SPOTs membrane was first blocked with 5% nonfat milk in TBS-T (0.1 M TrisHCl (pH 7.4), 150 mM NaCl, and 0.1% Tween 20) overnight at 4°C. GST alone or GST fusion proteins (0.25 μM) were incubated with the SPOTs membrane in the same buffer containing 1 mM DTT for 1Â½ hours at room temperature and then washed with TBS-T. Anti-GST (Amersham) antibodies were used to detect GST fusion proteins and then incubated with anti-Goat Alexa-Fluor-680 (Molecular Probes). The array membrane was subsequently washed four times with TBS-T for 10 min. Peptides that bound the domain of interest were visualized by Li-Cor Odyssey using the 700 nm channel. Intensities were calculated using a grid with 192 circular features of 2 mm diameter, each centered around a peptide spot to avoid scoring SPOTs with halo or rings. For each feature, the average (integrated) intensity was used for downstream analysis.

#### Fluorescence polarization

Peptides were synthesized using FMOC-chemistry onto pre-loaded tenta-gel resins. Peptides were then labeled with Rhodamine B (Abbey Color) and then cleaved using trifluoroacetic acid. Peptides were lyophilized and then purified using a LC/MS (Agilent 2100). Dissociation constants were measured using the Beacon 2000 (Invitrogen) as previously described [40].

#### Data analysis

All analysis steps were performed as previously described [86]. Peptide intensity scores (excluding those defined as non-specific) were averaged across each 192-peptide array, producing an array mean. Array-positive binding was ascribed to interactions with intensities greater than three times the array mean. Peptide spots with average intensity values between 1X-3X the array mean were defined as ‘indeterminate’. Those with intensities below 1X mean were defined as array negative. Non-specific signal was detected by arraying three separate 192 arrays with three separate GST preps at 0.25 μM. Non-specific binding peptides were identified as those with signal intensities greater than 3X the array mean in at least two of three trials.

#### Phosphorylation status and solvent exposed tyrosines

The structures files of InsR (1IRK, 2DTG), IGF-1R (1P4O), IRS-1 (1IRS, 1QQG), FGFR1 (1FGK), FGFR2 (2PVF), FRS2 (1XR0), p62DOK1 (2 V76), PLCG1 (1HSQ, 2HSP) collected from Protein Data Bank (PBD) (http://www.rcsb.org). Surface accessible tyrosines were solved using the Gerstein algorithm (http://helixweb.nih.gov/structbio/). The phosphorylation status of the 192 sites was identified using the protein modification resource, Phosphosite (http://www.phosphosite.org).

#### PSSMs and EDSM

For each SH2 domain a position specific scoring matrix (PSSM) was calculated for the array-positive peptides (posPSSM). A second PSSM was calculated for all peptides, excluding those judged to be non-specific, as the expected distribution of amino acids represented on the array (exPSSM). Subtracting exPSSM from posPSSM yields the expectation deviation scoring matrix or EDSM. The EDSM for each SH2 domain was visualized as a logo of positive and negative factors using Weblogo [69].

#### EDSM clustering

The unbiased position specific expectation deviation scoring matrix was expanded into a hyper-dimensional vector representation, and the Euclidean distances between vectors was computed. The resulting N-by-N distance matrix was then clustered using the Fitch-Margoliash method in the Phylip package [85]. The unrooted tree was drawn using the MEGA package [86].

#### Reported interactions

Reported peptide interactions were collected by searching HPRD and literature. Reported protein interactions were collected from the major protein-protein interaction databases of MINT [50], BIND [51], HPRD [52], and DIP [53] using UniHI [49].

#### Cells lines and GST-pull downs

Chinese Hamster Ovary (CHO) cells stably overexpressing insulin receptor (InsR) and IRS-1 were graciously provided by Xiao Jian Sun (UChicago). CHO cells were grown in DMEM/F12 supplemented with 10% fetal bovine serum, penicillin and streptomycin. CHO cells were serum starved for 24 hours and treated with and without insulin (100 nM) for 5 mins. Cells were lysed in HNTG (20 mM Hepes 7.5, NaCl, 1% Triton X-100, 10% Glycerol, 1 mM NaV0_4_) with protease inhibitors (1 mM PMSF, aprotonin and leupeptin). Pre-cleared lysates were incubated with GST-SH2 domains immobilized on glutathione beads and rocked for 3 hours at 4°C. Activated InsR and IRS-1 were detected using anti-phosphotyrosine 4 G10 (Upstate).

## Abbreviations

SH: Src Homology; Ins: Insulin; IGF: Insulin Growth Factor; FGF: Fibroblast Growth Factor; HPRD: Human Protein Resource Database; PTK: Protein Tyrosine Kinase; RTK: Receptor Tyrosine Kinase; PSSM: Position Specific Scoring Matrix.

## Competing interests

Both authors declare that they have no competing interests.

## Authors’ contributions

BAL and PDN designed the study and drafted the manuscript; BAL, BWE, KH, and ABS conducted the experiments. BAL, KJ and PDN developed the methodologies and bioinformatic analysis. BAL, BWE, and ABS provided analysis of the expression data. All authors read and approved the final manuscript.

## Supplementary Material

Additional file 1 **This table includes the position of the peptide on the array, peptide sequence, protein name, site of tyrosine phosphorylation and the status of phosphorylation based on Phosphosite (**http://www.phosphosite.org**).** Information regarding whether the peptide spot is considered “non-specific” is indicated. SH2 domains that bound >3× and between 2× and 3× the mean are also listed. Click here for file

Additional file 2 Supplementary materials includes a detailed description of GST background removal, supplemental tables and supplemental figures. Click here for file

Additional file 3 Complete list of SH2 domains tested onto the SPOT arrays. Click here for file

## References

[B1] van der Geer P Hunter T Lindberg RA Receptor protein-tyrosine kinases and their signal transduction pathways Annu Rev Cell Biol 1994 10 251 337 7888178 10.1146/annurev.cb.10.110194.001343

[B2] Fantl WJ Johnson DE Williams LT Signalling by receptor tyrosine kinases Annu Rev Biochem 1993 62 453 481 7688944 10.1146/annurev.bi.62.070193.002321

[B3] Lemmon MA Schlessinger J Cell signaling by receptor tyrosine kinases Cell 2010 141 1117 1134 20602996 10.1016/j.cell.2010.06.011 PMC2914105

[B4] Yaffe MB Phosphotyrosine-binding domains in signal transduction Nat Rev Mol Cell Biol 2002 3 177 186 11994738 10.1038/nrm759

[B5] Pawson T Specificity in signal transduction: from phosphotyrosine-SH2 domain interactions to complex cellular systems Cell 2004 116 191 203 14744431 10.1016/s0092-8674(03)01077-8

[B6] Pawson T Gish GD Nash P SH2 domains, interaction modules and cellular wiring Trends Cell Biol 2001 11 504 511 11719057 10.1016/s0962-8924(01)02154-7

[B7] Matsuda M Mayer BJ Fukui Y Hanafusa H Binding of transforming protein, P47gag-crk, to a broad range of phosphotyrosine-containing proteins Science 1990 248 1537 1539 1694307 10.1126/science.1694307

[B8] Margolis B Li N Koch A Mohammadi M Hurwitz DR Zilberstein A Ullrich A Pawson T Schlessinger J The tyrosine phosphorylated carboxyterminus of the EGF receptor is a binding site for GAP and PLC-gamma EMBO J 1990 9 4375 4380 2176151 10.1002/j.1460-2075.1990.tb07887.x PMC552227

[B9] Moran MF Koch CA Anderson D Ellis C England L Martin GS Pawson T Src homology region 2 domains direct protein-protein interactions in signal transduction Proc Natl Acad Sci USA 1990 87 8622 8626 2236073 10.1073/pnas.87.21.8622 PMC55009

[B10] Koch CA Anderson D Moran MF Ellis C Pawson T SH2 and SH3 domains: elements that control interactions of cytoplasmic signaling proteins Science 1991 252 668 674 1708916 10.1126/science.1708916

[B11] Liu BA Engelmann BW Nash PD The language of SH2 domain interactions defines phosphotyrosine-mediated signal transduction FEBS Lett 2012 586 2597 2605 22569091 10.1016/j.febslet.2012.04.054

[B12] Pawson T Nash P Assembly of cell regulatory systems through protein interaction domains Science 2003 300 445 452 12702867 10.1126/science.1083653

[B13] Pawson T Nash P Protein-protein interactions define specificity in signal transduction Genes Dev 2000 14 1027 1047 10809663

[B14] Liu BA Jablonowski K Raina M Arce M Pawson T Nash PD The Human and Mouse Complement of SH2 Domain Proteins-Establishing the Boundaries of Phosphotyrosine Signaling Mol Cell 2006 22 851 868 16793553 10.1016/j.molcel.2006.06.001

[B15] Liu BA Shah E Jablonowski K Stergachis A Engelmann BW Nash PD The SH2 domain-containing proteins in 21 extant species establish the provenance and scope of phosphotyrosine signaling in Eukaryotes Sci Signal 2011 4 ra83 22155787 10.1126/scisignal.2002105 PMC4255630

[B16] Lappalainen I Thusberg J Shen B Vihinen M Genome wide analysis of pathogenic SH2 domain mutations Proteins 2008 72 779 792 18260110 10.1002/prot.21970

[B17] Bradshaw JM Mitaxov V Waksman G Investigation of phosphotyrosine recognition by the SH2 domain of the Src kinase J Mol Biol 1999 293 971 985 10543978 10.1006/jmbi.1999.3190

[B18] Bradshaw JM Waksman G Molecular recognition by SH2 domains Adv Protein Chem 2002 61 161 210 12461824 10.1016/s0065-3233(02)61005-8

[B19] Songyang Z Shoelson SE Chaudhuri M Gish G Pawson T Haser WG King F Roberts T Ratnofsky S Lechleider RJ SH2 domains recognize specific phosphopeptide sequences Cell 1993 72 767 778 7680959 10.1016/0092-8674(93)90404-e

[B20] Huang H Li L Wu C Schibli D Colwill K Ma S Li C Roy P Ho K Songyang Z Defining the specificity space of the human SRC homology 2 domain Mol Cell Proteomics 2008 7 768 784 17956856 10.1074/mcp.M700312-MCP200

[B21] Obenauer JC Cantley LC Yaffe MB Scansite 2.0: Proteome-wide prediction of cell signaling interactions using short sequence motifs Nucleic Acids Res 2003 31 3635 3641 12824383 10.1093/nar/gkg584 PMC168990

[B22] Liu BA Jablonowski K Shah EE Engelmann BW Jones RB Nash PD SH2 domains recognize contextual peptide sequence information to determine selectivity Mol Cell Proteomics 2010 9 2391 2404 20627867 10.1074/mcp.M110.001586 PMC2984226

[B23] Machida K Thompson CM Dierck K Jablonowski K Karkkainen S Liu B Zhang H Nash PD Newman DK Nollau P High-throughput phosphotyrosine profiling using SH2 domains Mol Cell 2007 26 899 915 17588523 10.1016/j.molcel.2007.05.031

[B24] Dierck K Machida K Voigt A Thimm J Horstmann M Fiedler W Mayer BJ Nollau P Quantitative multiplexed profiling of cellular signaling networks using phosphotyrosine-specific DNA-tagged SH2 domains Nat Methods 2006 3 737 744 16929320 10.1038/nmeth917

[B25] Machida K Mayer BJ The SH2 domain: versatile signaling module and pharmaceutical target Biochim Biophys Acta 2005 1747 1 25 15680235 10.1016/j.bbapap.2004.10.005

[B26] Machida K Mayer BJ Nollau P Profiling the global tyrosine phosphorylation state Mol Cell Proteomics 2003 2 215 233 12754303 10.1074/mcp.R300002-MCP200

[B27] Machida K Eschrich S Li J Bai Y Koomen J Mayer BJ Haura EB Characterizing tyrosine phosphorylation signaling in lung cancer using SH2 profiling PLoS One 2010 5 e13470 20976048 10.1371/journal.pone.0013470 PMC2957407

[B28] Vetter SW Zhang ZY Probing the phosphopeptide specificities of protein tyrosine phosphatases, SH2 and PTB domains with combinatorial library methods Curr Protein Pept Sci 2002 3 365 397 12370002 10.2174/1389203023380594

[B29] Songyang Z Shoelson SE McGlade J Olivier P Pawson T Bustelo XR Barbacid M Sabe H Hanafusa H Yi T Specific motifs recognized by the SH2 domains of Csk, 3BP2, fps/fes, GRB-2, HCP, SHC, Syk, and Vav Mol Cell Biol 1994 14 2777 2785 7511210 10.1128/mcb.14.4.2777-2785.1994 PMC358643

[B30] Rodriguez M Li SS Harper JW Songyang Z An oriented peptide array library (OPAL) strategy to study protein-protein interactions J Biol Chem 2004 279 8802 8807 14679191 10.1074/jbc.M311886200

[B31] Cochrane D Webster C Masih G McCafferty J Identification of natural ligands for SH2 domains from a phage display cDNA library J Mol Biol 2000 297 89 97 10704309 10.1006/jmbi.2000.3561

[B32] Panni S Montecchi-Palazzi L Kiemer L Cabibbo A Paoluzi S Santonico E Landgraf C Volkmer-Engert R Bachi A Castagnoli L Cesareni G Combining peptide recognition specificity and context information for the prediction of the 14–3–3-mediated interactome in S. cerevisiae and H. sapiens Proteomics 2011 11 128 143 21182200 10.1002/pmic.201000030

[B33] Huttlin EL Jedrychowski MP Elias JE Goswami T Rad R Beausoleil SA Villen J Haas W Sowa ME Gygi SP A tissue-specific atlas of mouse protein phosphorylation and expression Cell 2010 143 1174 1189 21183079 10.1016/j.cell.2010.12.001 PMC3035969

[B34] Hlavacek WS Faeder JR The complexity of cell signaling and the need for a new mechanics Sci Signal 2009 2 pe46 19638613 10.1126/scisignal.281pe46

[B35] Yang J Hlavacek WS Scaffold-mediated nucleation of protein signaling complexes: Elementary principles Math Biosci 2011 232 164 173 21683720 10.1016/j.mbs.2011.06.003 PMC3137898

[B36] Uhlen M Oksvold P Fagerberg L Lundberg E Jonasson K Forsberg M Zwahlen M Kampf C Wester K Hober S Towards a knowledge-based Human Protein Atlas Nat Biotechnol 2010 28 1248 1250 21139605 10.1038/nbt1210-1248

[B37] Weiser AA Or-Guil M Tapia V Leichsenring A Schuchhardt J Frommel C Volkmer-Engert R SPOT synthesis: reliability of array-based measurement of peptide binding affinity Anal Biochem 2005 342 300 311 15950918 10.1016/j.ab.2005.04.033

[B38] Katz C Levy-Beladev L Rotem-Bamberger S Rito T Rudiger SG Friedler A Studying protein-protein interactions using peptide arrays Chem Soc Rev 2011 40 2131 2145 21243154 10.1039/c0cs00029a

[B39] Kramer A Reineke U Dong L Hoffmann B Hoffmuller U Winkler D Volkmer-Engert R Schneider-Mergener J Spot synthesis: observations and optimizations J Pept Res 1999 54 319 327 10532237 10.1034/j.1399-3011.1999.00108.x

[B40] Nash P Tang X Orlicky S Chen Q Gertler FB Mendenhall MD Sicheri F Pawson T Tyers M Multisite phosphorylation of a CDK inhibitor sets a threshold for the onset of DNA replication Nature 2001 414 514 521 11734846 10.1038/35107009

[B41] Frank R The SPOT-synthesis technique. Synthetic peptide arrays on membrane supports--principles and applications J Immunol Methods 2002 267 13 26 12135797 10.1016/s0022-1759(02)00137-0

[B42] Frank R Overwin H SPOT synthesis. Epitope analysis with arrays of synthetic peptides prepared on cellulose membranes Methods Mol Biol 1996 66 149 169 8959713 10.1385/0-89603-375-9:149

[B43] Li SC Gish G Yang D Coffey AJ Forman-Kay JD Ernberg I Kay LE Pawson T Novel mode of ligand binding by the SH2 domain of the human XLP disease gene product SAP/SH2D1A Curr Biol 1999 9 1355 1362 10607564 10.1016/s0960-9822(00)80080-9

[B44] Mahrenholz CC Tapia V Stigler RD Volkmer R A study to assess the cross-reactivity of cellulose membrane-bound peptides with detection systems: an analysis at the amino acid level J Pept Sci 2010 16 297 302 20474041 10.1002/psc.1237

[B45] Myers MG Jr Backer JM Sun XJ Shoelson S Hu P Schlessinger J Yoakim M Schaffhausen B White MF IRS-1 activates phosphatidylinositol 3'-kinase by associating with src homology 2 domains of p85 Proc Natl Acad Sci USA 1992 89 10350 10354 1332046 10.1073/pnas.89.21.10350 PMC50336

[B46] Sun XJ Pons S Asano T Myers MG Jr Glasheen E White MF The Fyn tyrosine kinase binds Irs-1 and forms a distinct signaling complex during insulin stimulation J Biol Chem 1996 271 10583 10587 8631859 10.1074/jbc.271.18.10583

[B47] Myers MG Jr Zhang Y Aldaz GA Grammer T Glasheen EM Yenush L Wang LM Sun XJ Blenis J Pierce JH White MF YMXM motifs and signaling by an insulin receptor substrate 1 molecule without tyrosine phosphorylation sites Mol Cell Biol 1996 16 4147 4155 8754813 10.1128/mcb.16.8.4147 PMC231411

[B48] Sadowski I Stone JC Pawson T A noncatalytic domain conserved among cytoplasmic protein-tyrosine kinases modifies the kinase function and transforming activity of Fujinami sarcoma virus P130gag-fps Mol Cell Biol 1986 6 4396 4408 3025655 10.1128/mcb.6.12.4396-4408.1986 PMC367222

[B49] Chaurasia G Iqbal Y Hanig C Herzel H Wanker EE Futschik ME UniHI: an entry gate to the human protein interactome Nucleic Acids Res 2007 35 D590 D594 17158159 10.1093/nar/gkl817 PMC1781159

[B50] Chatr-aryamontri A Ceol A Palazzi LM Nardelli G Schneider MV Castagnoli L Cesareni G MINT: the Molecular INTeraction database Nucleic Acids Res 2007 35 D572 D574 17135203 10.1093/nar/gkl950 PMC1751541

[B51] Bader GD Betel D Hogue CW BIND: the Biomolecular Interaction Network Database Nucleic Acids Res 2003 31 248 250 12519993 10.1093/nar/gkg056 PMC165503

[B52] Mishra GR Suresh M Kumaran K Kannabiran N Suresh S Bala P Shivakumar K Anuradha N Reddy R Raghavan TM Human protein reference database–2006 update Nucleic Acids Res 2006 34 D411 D414 16381900 10.1093/nar/gkj141 PMC1347503

[B53] Salwinski L Miller CS Smith AJ Pettit FK Bowie JU Eisenberg D The Database of Interacting Proteins: 2004 update Nucleic Acids Res 2004 32 D449 D451 14681454 10.1093/nar/gkh086 PMC308820

[B54] Liu BA Engelmann BW Nash PD High-throughput analysis of peptide binding modules Proteomics 2012 12 1527 1546 22610655 10.1002/pmic.201100599 PMC4255589

[B55] Mohammadi M Honegger AM Rotin D Fischer R Bellot F Li W Dionne CA Jaye M Rubinstein M Schlessinger J A tyrosine-phosphorylated carboxy-terminal peptide of the fibroblast growth factor receptor (Flg) is a binding site for the SH2 domain of phospholipase C-gamma 1 Mol Cell Biol 1991 11 5068 5078 1656221 10.1128/mcb.11.10.5068-5078.1991 PMC361508

[B56] Peters KG Marie J Wilson E Ives HE Escobedo J Del Rosario M Mirda D Williams LT Point mutation of an FGF receptor abolishes phosphatidylinositol turnover and Ca2+ flux but not mitogenesis Nature 1992 358 678 681 1379697 10.1038/358678a0

[B57] Kaushansky A Gordus A Chang B Rush J MacBeath G A quantitative study of the recruitment potential of all intracellular tyrosine residues on EGFR, FGFR1 and IGF1R Mol Biosyst 2008 4 643 653 18493663 10.1039/b801018h PMC2811368

[B58] Jones RB Gordus A Krall JA MacBeath G A quantitative protein interaction network for the ErbB receptors using protein microarrays Nature 2006 439 168 174 16273093 10.1038/nature04177

[B59] Bock I Kudithipudi S Tamas R Kungulovski G Dhayalan A Jeltsch A Application of Celluspots peptide arrays for the analysis of the binding specificity of epigenetic reading domains to modified histone tails BMC Biochem 2011 12 48 21884582 10.1186/1471-2091-12-48 PMC3176149

[B60] Stiffler MA Chen JR Grantcharova VP Lei Y Fuchs D Allen JE Zaslavskaia LA MacBeath G PDZ domain binding selectivity is optimized across the mouse proteome Science 2007 317 364 369 17641200 10.1126/science.1144592 PMC2674608

[B61] Kaushansky A Allen JE Gordus A Stiffler MA Karp ES Chang BH MacBeath G Quantifying protein-protein interactions in high throughput using protein domain microarrays Nat Protoc 2010 5 773 790 20360771 10.1038/nprot.2010.36 PMC3085283

[B62] Chang BH Gujral TS Karp ES BuKhalid R Grantcharova VP MacBeath G A systematic family-wide investigation reveals that 30% of mammalian PDZ domains engage in PDZ-PDZ interactions Chem Biol 2011 18 1143 1152 21944753 10.1016/j.chembiol.2011.06.013 PMC3442787

[B63] Liu SK Fang N Koretzky GA McGlade CJ The hematopoietic-specific adaptor protein gads functions in T-cell signaling via interactions with the SLP-76 and LAT adaptors Curr Biol 1999 9 67 75 10021361 10.1016/s0960-9822(99)80017-7

[B64] Feng GS Ouyang YB Hu DP Shi ZQ Gentz R Ni J Grap is a novel SH3-SH2-SH3 adaptor protein that couples tyrosine kinases to the Ras pathway J Biol Chem 1996 271 12129 12132 8647802 10.1074/jbc.271.21.12129

[B65] Hornbeck PV Chabra I Kornhauser JM Skrzypek E Zhang B PhosphoSite: A bioinformatics resource dedicated to physiological protein phosphorylation Proteomics 2004 4 1551 1561 15174125 10.1002/pmic.200300772

[B66] Videlock EJ Chung VK Hall JM Hines J Agapakis CM Austin DJ Identification of a molecular recognition role for the activation loop phosphotyrosine of the SRC tyrosine kinase J Am Chem Soc 2005 127 1600 1601 15700969 10.1021/ja047957c

[B67] Gerstein M Chothia C Packing at the protein-water interface Proc Natl Acad Sci USA 1996 93 10167 10172 8816770 10.1073/pnas.93.19.10167 PMC38355

[B68] Tsai J Taylor R Chothia C Gerstein M The packing density in proteins: standard radii and volumes J Mol Biol 1999 290 253 266 10388571 10.1006/jmbi.1999.2829

[B69] Crooks GE Hon G Chandonia JM Brenner SE WebLogo: a sequence logo generator Genome Res 2004 14 1188 1190 15173120 10.1101/gr.849004 PMC419797

[B70] Liu BA Nash PD Evolution of SH2 domains and phosphotyrosine signaling networks Philos Trans R Soc Lond B Biol Sci 2012 367 2555 2572 10.1098/rstb.2012.0107 PMC3415846 22889907

[B71] Bisson N James DA Ivosev G Tate SA Bonner R Taylor L Pawson T Selected reaction monitoring mass spectrometry reveals the dynamics of signaling through the GRB2 adaptor Nat Biotechnol 2011 29 653 658 21706016 10.1038/nbt.1905

[B72] Gallagher EJ LeRoith D The proliferating role of insulin and insulin-like growth factors in cancer Trends Endocrinol Metab 2010 21 610 618 20663687 10.1016/j.tem.2010.06.007 PMC2949481

[B73] Siddle K Signalling by insulin and IGF receptors: supporting acts and new players J Mol Endocrinol 2011 47 R1 R10 21498522 10.1530/JME-11-0022

[B74] Heidegger I Pircher A Klocker H Massoner P Targeting the insulin-like growth factor network in cancer therapy Cancer Biol Ther 2011 11 701 707 21311212 10.4161/cbt.11.8.14689

[B75] Pansuria M Xi H Li L Yang XF Wang H Insulin resistance, metabolic stress, and atherosclerosis Front Biosci (Schol Ed) 2012 4 916 931 22202099 10.2741/s308 PMC3319745

[B76] Kim B Feldman EL Insulin resistance in the nervous system Trends Endocrinol Metab 2012 23 133 141 22245457 10.1016/j.tem.2011.12.004 PMC3392648

[B77] Bradshaw JM Waksman G Calorimetric examination of high-affinity Src SH2 domain-tyrosyl phosphopeptide binding: dissection of the phosphopeptide sequence specificity and coupling energetics Biochemistry 1999 38 5147 5154 10213620 10.1021/bi982974y

[B78] Lemmon MA Ladbury JE Mandiyan V Zhou M Schlessinger J Independent binding of peptide ligands to the SH2 and SH3 domains of Grb2 J Biol Chem 1994 269 31653 31658 7527391

[B79] Piccione E Case RD Domchek SM Hu P Chaudhuri M Backer JM Schlessinger J Shoelson SE Phosphatidylinositol 3-kinase p85 SH2 domain specificity defined by direct phosphopeptide/SH2 domain binding Biochemistry 1993 32 3197 3202 8384875 10.1021/bi00064a001

[B80] Hunter T Cooper JA Protein-tyrosine kinases Annu Rev Biochem 1985 54 897 930 2992362 10.1146/annurev.bi.54.070185.004341

[B81] Peri S Navarro JD Kristiansen TZ Amanchy R Surendranath V Muthusamy B Gandhi TK Chandrika KN Deshpande N Suresh S Human protein reference database as a discovery resource for proteomics Nucleic Acids Res 2004 32 D497 D501 14681466 10.1093/nar/gkh070 PMC308804

[B82] Linding R Jensen LJ Ostheimer GJ van Vugt MA Jorgensen C Miron IM Diella F Colwill K Taylor L Elder K Systematic discovery of in vivo phosphorylation networks Cell 2007 129 1415 1426 17570479 10.1016/j.cell.2007.05.052 PMC2692296

[B83] Zarrinpar A Park SH Lim WA Optimization of specificity in a cellular protein interaction network by negative selection Nature 2003 426 676 680 14668868 10.1038/nature02178

[B84] Mayer BJ Hamaguchi M Hanafusa H A novel viral oncogene with structural similarity to phospholipase C Nature 1988 332 272 275 2450282 10.1038/332272a0

[B85] Felsenstein J PHYLIP -- Phylogeny Inference Package (Version 3.2) Cladistics 1989 5 164 166

[B86] Tamura K Dudley J Nei M Kumar S MEGA4: Molecular Evolutionary Genetics Analysis (MEGA) software version 4.0 Mol Biol Evol 2007 24 1596 1599 17488738 10.1093/molbev/msm092

[B87] Shin NY Dise RS Schneider-Mergener J Ritchie MD Kilkenny DM Hanks SK Subsets of the major tyrosine phosphorylation sites in Crk-associated substrate (CAS) are sufficient to promote cell migration J Biol Chem 2004 279 38331 38337 15247284 10.1074/jbc.M404675200

[B88] Sawasdikosol S Chang JH Pratt JC Wolf G Shoelson SE Burakoff SJ Tyrosine-phosphorylated Cbl binds to Crk after T cell activation J Immunol 1996 157 110 116 8683103

[B89] Howlett CJ Bisson SA Resek ME Tigley AW Robbins SM The proto-oncogene p120(Cbl) is a downstream substrate of the Hck protein-tyrosine kinase Biochem Biophys Res Commun 1999 257 129 138 10092522 10.1006/bbrc.1999.0427

[B90] Hunter S Burton EA Wu SC Anderson SM Fyn associates with Cbl and phosphorylates tyrosine 731 in Cbl, a binding site for phosphatidylinositol 3-kinase J Biol Chem 1999 274 2097 2106 9890970 10.1074/jbc.274.4.2097

[B91] Frese S Schubert WD Findeis AC Marquardt T Roske YS Stradal TE Heinz DW The phosphotyrosine peptide binding specificity of Nck1 and Nck2 Src homology 2 domains J Biol Chem 2006 281 18236 18245 16636066 10.1074/jbc.M512917200

[B92] Skolnik EY Lee CH Batzer A Vicentini LM Zhou M Daly R Myers MJ Jr Backer JM Ullrich A White MF The SH2/SH3 domain-containing protein GRB2 interacts with tyrosine-phosphorylated IRS1 and Shc: implications for insulin control of ras signalling EMBO J 1993 12 1929 1936 8491186 10.1002/j.1460-2075.1993.tb05842.x PMC413414

[B93] Rozakis-Adcock M McGlade J Mbamalu G Pelicci G Daly R Li W Batzer A Thomas S Brugge J Pelicci PG Association of the Shc and Grb2/Sem5 SH2-containing proteins is implicated in activation of the Ras pathway by tyrosine kinases Nature 1992 360 689 692 1465135 10.1038/360689a0

[B94] Muller K Gombert FO Manning U Grossmuller F Graff P Zaegel H Zuber JF Freuler F Tschopp C Baumann G Rapid identification of phosphopeptide ligands for SH2 domains. Screening of peptide libraries by fluorescence-activated bead sorting J Biol Chem 1996 271 16500 16505 8663178

[B95] Ottinger EA Botfield MC Shoelson SE Tandem SH2 domains confer high specificity in tyrosine kinase signaling J Biol Chem 1998 273 729 735 9422724 10.1074/jbc.273.2.729

[B96] Pascal SM Singer AU Gish G Yamazaki T Shoelson SE Pawson T Kay LE Forman-Kay JD Nuclear magnetic resonance structure of an SH2 domain of phospholipase C-gamma 1 complexed with a high affinity binding peptide Cell 1994 77 461 472 8181064 10.1016/0092-8674(94)90160-0

[B97] Finerty PJ Jr Mittermaier AK Muhandiram R Kay LE Forman-Kay JD NMR dynamics-derived insights into the binding properties of a peptide interacting with an SH2 domain Biochemistry 2005 44 694 703 15641795 10.1021/bi048641k

[B98] Malabarba MG Milia E Faretta M Zamponi R Pelicci PG Di Fiore PP A repertoire library that allows the selection of synthetic SH2s with altered binding specificities Oncogene 2001 20 5186 5194 11526507 10.1038/sj.onc.1204654

[B99] Moores SL Selfors LM Fredericks J Breit T Fujikawa K Alt FW Brugge JS Swat W Vav family proteins couple to diverse cell surface receptors Mol Cell Biol 2000 20 6364 6373 10938113 10.1128/mcb.20.17.6364-6373.2000 PMC86111

[B100] Morra M Lu J Poy F Martin M Sayos J Calpe S Gullo C Howie D Rietdijk S Thompson A Structural basis for the interaction of the free SH2 domain EAT-2 with SLAM receptors in hematopoietic cells EMBO J 2001 20 5840 5852 11689425 10.1093/emboj/20.21.5840 PMC125701

[B101] Zhou MM Meadows RP Logan TM Yoon HS Wade WS Ravichandran KS Burakoff SJ Fesik SW Solution structure of the Shc SH2 domain complexed with a tyrosine-phosphorylated peptide from the T-cell receptor Proc Natl Acad Sci USA 1995 92 7784 7788 7544002 10.1073/pnas.92.17.7784 PMC41230

[B102] Symes A Stahl N Reeves SA Farruggella T Servidei T Gearan T Yancopoulos G Fink JS The protein tyrosine phosphatase SHP-2 negatively regulates ciliary neurotrophic factor induction of gene expression Curr Biol 1997 7 697 700 9285712 10.1016/s0960-9822(06)00298-3

[B103] Yeh RH Lee TR Lawrence DS From consensus sequence peptide to high affinity ligand, a "library scan" strategy J Biol Chem 2001 276 12235 12240 11278862 10.1074/jbc.M011232200

[B104] Boomer JS Tan TH Functional interactions of HPK1 with adaptor proteins J Cell Biochem 2005 95 34 44 15770651 10.1002/jcb.20401

[B105] Kayali AG Eichhorn J Haruta T Morris AJ Nelson JG Vollenweider P Olefsky JM Webster NJ Association of the insulin receptor with phospholipase C-gamma (PLCgamma) in 3 T3-L1 adipocytes suggests a role for PLCgamma in metabolic signaling by insulin J Biol Chem 1998 273 13808 13818 9593725 10.1074/jbc.273.22.13808

[B106] Lee CH Li W Nishimura R Zhou M Batzer AG Myers MG Jr White MF, Schlessinger J, Skolnik EY: Nck associates with the SH2 domain-docking protein IRS-1 in insulin-stimulated cells Proc Natl Acad Sci USA 1993 90 11713 11717 8265614 10.1073/pnas.90.24.11713 PMC48054

[B107] Case RD Piccione E Wolf G Benett AM Lechleider RJ Neel BG Shoelson SE SH-PTP2/Syp SH2 domain binding specificity is defined by direct interactions with platelet-derived growth factor beta-receptor, epidermal growth factor receptor, and insulin receptor substrate-1-derived phosphopeptides J Biol Chem 1994 269 10467 10474 8144631

[B108] Sun XJ Crimmins DL Myers MG Jr Miralpeix M White MF Pleiotropic insulin signals are engaged by multisite phosphorylation of IRS-1 Mol Cell Biol 1993 13 7418 7428 7504175 10.1128/mcb.13.12.7418-7428.1993 PMC364813

[B109] Skolnik EY Batzer A Li N Lee CH Lowenstein E Mohammadi M Margolis B Schlessinger J The function of GRB2 in linking the insulin receptor to Ras signaling pathways Science 1993 260 1953 1955 8316835 10.1126/science.8316835

[B110] Myers MG Jr Mendez R Shi P Pierce JH Rhoads R White MF The COOH-terminal tyrosine phosphorylation sites on IRS-1 bind SHP-2 and negatively regulate insulin signaling J Biol Chem 1998 273 26908 26914 9756938 10.1074/jbc.273.41.26908

[B111] Sozzani P Hasan L Seguelas MH Caput D Ferrara P Pipy B Cambon C IL-13 induces tyrosine phosphorylation of phospholipase C gamma-1 following IRS-2 association in human monocytes: relationship with the inhibitory effect of IL-13 on ROI production Biochem Biophys Res Commun 1998 244 665 670 9535722 10.1006/bbrc.1998.8314

[B112] Patti ME Sun XJ Bruening JC Araki E Lipes MA White MF Kahn CR 4PS/insulin receptor substrate (IRS)-2 is the alternative substrate of the insulin receptor in IRS-1-deficient mice J Biol Chem 1995 270 24670 24673 7559579 10.1074/jbc.270.42.24670

[B113] Cross MJ Lu L Magnusson P Nyqvist D Holmqvist K Welsh M Claesson-Welsh L The Shb adaptor protein binds to tyrosine 766 in the FGFR-1 and regulates the Ras/MEK/MAPK pathway via FRS2 phosphorylation in endothelial cells Mol Biol Cell 2002 13 2881 2893 12181353 10.1091/mbc.E02-02-0103 PMC117949

[B114] Kong M Wang CS Donoghue DJ Interaction of fibroblast growth factor receptor 3 and the adapter protein SH2-B. A role in STAT5 activation J Biol Chem 2002 277 15962 15970 11827956 10.1074/jbc.M102777200

[B115] Vainikka S Joukov V Wennstrom S Bergman M Pelicci PG Alitalo K Signal transduction by fibroblast growth factor receptor-4 (FGFR-4). Comparison with FGFR-1 J Biol Chem 1994 269 18320 18326 7518429

[B116] Kouhara H Hadari YR Spivak-Kroizman T Schilling J Bar-Sagi D Lax I Schlessinger J A lipid-anchored Grb2-binding protein that links FGF-receptor activation to the Ras/MAPK signaling pathway Cell 1997 89 693 702 9182757 10.1016/s0092-8674(00)80252-4

[B117] Hadari YR Kouhara H Lax I Schlessinger J Binding of Shp2 tyrosine phosphatase to FRS2 is essential for fibroblast growth factor-induced PC12 cell differentiation Mol Cell Biol 1998 18 3966 3973 9632781 10.1128/mcb.18.7.3966 PMC108981

[B118] Poulin B Sekiya F Rhee SG Intramolecular interaction between phosphorylated tyrosine-783 and the C-terminal Src homology 2 domain activates phospholipase C-gamma1 Proc Natl Acad Sci USA 2005 102 4276 4281 15764700 10.1073/pnas.0409590102 PMC555506

[B119] Schlaepfer DD Broome MA Hunter T Fibronectin-stimulated signaling from a focal adhesion kinase-c-Src complex: involvement of the Grb2, p130cas, and Nck adaptor proteins Mol Cell Biol 1997 17 1702 1713 9032297 10.1128/mcb.17.3.1702 PMC231895

[B120] Abassi YA Rehn M Ekman N Alitalo K Vuori K p130Cas Couples the tyrosine kinase Bmx/Etk with regulation of the actin cytoskeleton and cell migration J Biol Chem 2003 278 35636 35643 12832404 10.1074/jbc.M306438200

[B121] Nasertorabi F Tars K Becherer K Kodandapani R Liljas L Vuori K Ely KR Molecular basis for regulation of Src by the docking protein p130Cas J Mol Recognit 2006 19 30 38 16245368 10.1002/jmr.755

[B122] Kashige N Carpino N Kobayashi R Tyrosine phosphorylation of p62dok by p210bcr-abl inhibits RasGAP activity Proc Natl Acad Sci USA 2000 97 2093 2098 10688886 10.1073/pnas.040547997 PMC15759

[B123] Wick MJ Dong LQ Hu D Langlais P Liu F Insulin receptor-mediated p62dok tyrosine phosphorylation at residues 362 and 398 plays distinct roles for binding GTPase-activating protein and Nck and is essential for inhibiting insulin-stimulated activation of Ras and Akt J Biol Chem 2001 276 42843 42850 11551902 10.1074/jbc.M102116200

[B124] Lock P Casagranda F Dunn AR Independent SH2-binding sites mediate interaction of Dok-related protein with RasGTPase-activating protein and Nck J Biol Chem 1999 274 22775 22784 10428862 10.1074/jbc.274.32.22775

