# Supplementary material for: SRC Homology 2 Domain Binding Sites in Insulin, IGF-1 and FGF receptor mediated signaling networks reveal an extensive potential interactome
Source: Cell Commun Signal. 2012 Sep 14;10:27. doi: 10.1186/1478-811X-10-27 (PMC3514216; doi:10.1186/1478-811X-10-27)
Supplement: Additional file 1 — This table includes the position of the peptide on the array, peptide sequence, protein name, site of tyrosine phosphorylation and the status of phosphorylation based on Phosphosite (http://www.phosphosite.org). Information regarding whether the peptide spot is considered “non-specific” is indicated. SH2 domains that bound >3× and between 2× and 3× the mean are also listed. [file 1478-811X-10-27-S1.doc]

**Supplemental Materials**

**Src Homology 2 Domain Binding Partners in Insulin, IGF-1 and FGF Receptor Mediated Signaling Networks Reveal an Extensive Potential Interactome.**

Bernard A. Liu^1,2^, Karl Jablonowski^1^, Brett W. Engelmann^1,3^, Katherine Higginbotham^1^ and Piers D. Nash^1,2^*

^1^Ben May Department for Cancer Research and ^2^Committee on Cancer Biology and ^3^Biochemistry and Molecular Biology Department, The University of Chicago, Chicago, Illinois 60637

Running title: “SH2 Interactome for Ins, IGF-1 and FGF signaling”

Corresponding Author: Piers D. Nash; [pdnash.uchicago@gmail.com](mailto:pdnash.uchicago@gmail.com)

**Table of Contents**

Supplemental Sections

Supplemental Tables 3-5

Supplemental Figures 1-7

References

**Supplemental Sections**

**1. Elimination of background peptides**

To reduce the errors associated with identifying false-positives, non-specific interacting peptides (so-called ‘sticky’ peptides) were identified in two ways. Peptides that bound to GST, reflected by an intensity above the mean in two out of three separate trials, were tagged as non-specific binding peptides. Alternatively, peptides that exhibited above-mean binding to the majority of individual SH2 domains were used to corroborate an assessment of “non-specific”. This latter method relies upon the data-distribution properties obtained by probing each peptide with 50 SH2 domains and allows us to assess non-selective binding across a large panel of SH2 domains (**Figure S2**). We observe individual peptides falling into three categories. Peptides with specific interactions exhibit very low signals for most SH2 domains while exhibiting high signal intensities for those SH2 domains to which bound specifically, yielding a bimodal distribution with a first peak below 1X mean and a second peak above 3X mean (**Figure 2A**). Non-selective peptides exhibit above average binding across a wide spectrum of SH2 domains. This is illustrated in the binding histograms for each of the 192 peptides (**Figure S2B**). Peptides that failed to bind at all exhibit low LiCor signal intensity (**Figure 2C**). The distinction of the non-specific peptides was also noted using Transition Analysis approach (**Figure 2E**). Together, these analyses led to the removal of 40 non-specific peptides. We developed an Association Rule learning algorithm developed to identify patterns within non-specific peptides revealed that peptides containing tandem basic residues were most likely to result in non-specific binding [1, 2] (**Figure 2D**). The results of this analysis will aid in the development future addressable peptide arrays with lower rates of non-specific binding.

**Supplemental Tables**

**Table S1. SPOTS192 List (Excel File).**

This table includes the position of the peptide on the array, peptide sequence, protein name, site of tyrosine phosphorylation and the status of phosphorylation based on Phosphosite ([www.phosphosite.org](http://www.phosphosite.org/)). Information regarding whether the peptide spot is considered “non-specific” is indicated. SH2 domains that bound >3x and between 2x and 3x the mean are also listed. See the attached excel file.

**Table S2.** **Complete list of SH2 domains tested onto the SPOT arrays (Excel File).**

See the attached excel sheet for the compiled list of SH2 domains including the boundaries, species and expression details.

**Table S3. Comparison between Peptide arrays (P.A.) and SH2 protein microarrays (P.M.)**. (N.S. – Non-specific, N.D. – Not determined, N.B. – Non binding)

| **Peptide Sequence (* Cys replaced with Ala or Ser on peptide arrays)** | **Protein (Tyrosine phosphorylation site/SH2 binding site)** | **SH2 Domain** | **Peptide Array (X-mean)** | **Protein Microarray (Macbeath) – K_D_ (μM)** | **Fluorescence Polarization Asssay – K_D_ (μM)** | **Validation** | **Comments** |
| --- | --- | --- | --- | --- | --- | --- | --- |
| TPMLA**GVSEpYELPEDP**RD | FGFR1 Y463 | Brk | >3x | N.B. | 0.46 | Conflict, FP confirmed |  |
| TPMLA**GVSEpYELPEDP**RD | FGFR1 Y463 | Nck1 | >3x | N.B. | 2.45 |  |  |
| TPMLA**GVSEpYELPEDP**RD | FGFR1 Y463 | Grb2 | <1x | 0.34 | N.D. | Conflict | Does not conform to Grb2 consensus |
| TPMLA**GVSEpYELPEDP**RD | FGFR1 Y463 | Rasa1_N | >3x | 0.175 | 1.54 |  |  |
| TPMLA**GVSEpYELPEDP**RD | FGFR1 Y463 | Vav1 | >3x | N.B. | 1.7 | Conflict, FP confirmed |  |
| TPMLA**GVSEpYELPEDP**RD | FGFR1 Y463 | Yes | >3x | 0.45 | N.A | Positive (P.A and P.M) |  |
| TPMLA**GVSEpYELPEDP**RD | FGFR1 Y463 | Src | <3x | 0.33 | 6.85 | Conflict |  |
| TPMLA**GVSEpYELPEDP**RD | FGFR1 Y463 | Blk | btwn 2-3x | 0.15 | N.D. |  |  |
| TPMLA**GVSEpYELPEDP**RD | FGFR1 Y463 | Plcg2_N | >3x | N.B. | N.D. |  |  |
| TPMLA**GVSEpYELPEDP**RD | FGFR1 Y463 | Crk | <2x | N.B. | 44 | Both Negative |  |
| TPMLA**GVSEpYELPEDP**RD | FGFR1 Y463 | CrkL | <2x | N.B. | 50 | Both Negative |  |
| TPMLA**GVSEpYELPEDP**RD | FGFR1 Y463 | Itk | >3x | N.B. | 2.74 | Conflict |  |
| GACTQ**DGPLpYVIVEYA**SD | FGFR1 Y558 |  |  | N.D. | N.D. |  |  |
| DGPLY**VIVEpYASKGNL**RD | FGFR1 Y563 |  |  | N.D. | N.D. |  |  |
| YASKG**NLREpYLQARRP**PD | FGFR1 Y572 | N.S. | N.S. | N.D. | N.D. |  | Non-specific peptide (P.M.) |
| QARRP**PGLEpYCYNPSH**ND* | FGFR1 Y583 | Abl1 | >3x | 1.8 | N.D. | Positive (P.A. and P.M.) | Disulfide (Cys-Cys) interaction |
| QARRP**PGLEpYCYNPSH**ND* | FGFR1 Y583 | Abl2 | <2x | 0.34 |  | Conflict |  |
| QARRP**PGLEpYCYNPSH**ND* | FGFR1 Y583 | Itk | <2x | 0.97 |  | Conflict |  |
| QARRP**PGLEpYCYNPSH**ND* | FGFR1 Y583 | Yes | <2x | 1.55 |  | Conflict |  |
| QARRP**PGLEpYCYNPSH**ND* | FGFR1 Y583 | Grb2 | <2x | 1.62 |  | Conflict | Disulfide (Cys-Cys) interaction, Not Grb2 consensus (pYxNx) |
| QARRP**PGLEpYCYNPSH**ND* | FGFR1 Y583 | Grb7 | >3x | 1.00 |  | Positive |  |
| QARRP**PGLEpYCYNPSH**ND* | FGFR1 Y583 | CrkL | <2x | 1.55 |  | Conflict | Disulfide (Cys-Cys) interaction, Not CrkL consensus (pYxxP) |
| QARRP**PGLEpYCYNPSH**ND* | FGFR1 Y583 | Rasa1_N | <2x | 1.95 |  | Conflict |  |
| RRPPG**LEYCpYNPSHNP**ED | FGFR1 Y585 |  |  |  |  |  | Disulfide (Cys-Cys) interaction |
| SSKDL**VSCApYQVARGM**ED | FGFR1 Y605 | Abl2 | <2x | 0.012 | N.D | Conflict | Disulfide (Cys-Cys) interaction |
| SSKDL**VSCApYQVARGM**ED | FGFR1 Y605 | Crk | <2x | 0.26 |  | Conflict |  |
| SSKDL**VSCApYQVARGM**ED | FGFR1 Y605 | Crkl | <2x | 0.24 |  | Conflict |  |
| AYQVA**RGMEpYLASKKC**ID | FGFR1 Y613 | N.S. | N.S. | N.D. | N.D. |  | No peptide generated from |
| LARDI**HHIDpYYKKTTN**GD | FGFR1 Y653 | N.S. | N.S. | N.D. | N.D. |  | Peptide insoluble on Protein Microarrays |
| ARDIH**HIDYpYKKTTNG**RD | FGFR1 Y654 | Gads | <2x | 1.01 |  | Conflict |  |
| ARDIH**HIDYpYKKTTNG**RD | FGFR1 Y654 | Crk | <2x | 1.11 |  |  |  |
| APEAL**FDRIpYTHQSDV**WD | FGFR1 Y677 | N.S. | N.S. |  |  |  |  |
| EIFTL**GGSPpYPGVPVE**ED | FGFR1 Y701 | Abl2 | <2x | 0.40 |  | Conflict |  |
| EIFTL**GGSPpYPGVPVE**ED | FGFR1 Y701 | Src | <2x | 1.79 |  | Conflict |  |
| EIFTL**GGSPpYPGVPVE**ED | FGFR1 Y701 | Grb2 | <2x | 0.14 |  | Conflict |  |
| EIFTL**GGSPpYPGVPVE**ED | FGFR1 Y701 | Grb7 | <2x | 0.65 |  | Conflict |  |
| KPSNC**TNELpYMMMRDC**WD | FGFR1 Y730 |  |  |  |  |  | Disulfide (Cys-Cys) interaction |
| ALT**SNQEpYLDLSMD** | FGFR1 Y766 |  |  |  |  |  |  |
| LDLSM**PLDQpYSPSFPD**TD | FGFR1 Y776 |  |  | N.D. |  |  |  |

**Table S4. Exposed Tyrosines.**

Surface accessible tyrosines of the available structures ([www.rcsb.org](http://www.rcsb.org/)) were solved using the Gerstein algorithm (<http://helixweb.nih.gov/structbio/>).

| Protein | Tyrosine Site | Ang Sqd | Position Phosphorylated | Comments | RCSB File |
| --- | --- | --- | --- | --- | --- |
| PLCG1 | 802 | 54.545 | N/A |  | 2HSP |
| PLCG1 | 802 | 55.5 | N/A |  | 1HSQ |
| PLCG1 | 833 | 70.1 | No |  | 2HSP |
| PLCG1 | 833 | 61.715 | No |  | 1HSQ |
| PLCG1 | 845 | 78.53 | No |  | 2HSP |
| PLCG1 | 845 | 77.945 | N/A |  | 1HSQ |
| P130Cas | 12 | 90.875 | N/A |  | 1WYX |
| P130Cas | 67 | 110.505 | N/A |  | 1WYX |
| p62DOK1 | 174 | 12.98 | No |  | 2V76 |
| p62DOK1 | 203 | 13.9375 | No |  | 2V76 |
| p62DOK1 | 209 | 39.3675 | No |  | 2V76 |
| FRS2 | 48 | 77.98 | No |  | 1XR0 |
| FRS2 | 59 | 5.75 | No |  | 1XR0 |
| FRS2 | 65 | 2.31 | No |  | 1XR0 |
| FRS2 | 67 | 32.51 | No |  | 1XR0 |
| FGFR2 | 552 | 27.06 | No |  | 2PVF |
| FGFR2 | 557 | 62.64 | No |  | 2PVF |
| FGFR2 | 566 | 14.545 | No |  | 2PVF |
| FGFR2 | 587 | 176.5 | No |  | 2PVF |
| FGFR2 | 589 | 270.85 | No |  | 2PVF |
| FGFR2 | 609 | 35.855 | No |  | 2PVF |
| FGFR2 | 617 | 49.645 | No |  | 2PVF |
| FGFR2 | 657 | 103.365 | No | Activation Loop | 2PVF |
| FGFR2 | 658 | 167.75 | No | Activation Loop | 2PVF |
| FGFR2 | 681 | 3.535 | No |  | 2PVF |
| FGFR2 | 705 | 3.505 | No |  | 2PVF |
| FGFR2 | 734 | 26.79 | No |  | 2PVF |
| FGFR1 | 463 | 213.9 | Yes |  | 1FGK |
| FGFR1 | 558 | 29.45 | No |  | 1FGK |
| FGFR1 | 563 | 44.64 | No |  | 1FGK |
| FGFR1 | 572 | 13.48 | No |  | 1FGK |
| FGFR1 | 605 | 40.32 | Yes |  | 1FGK |
| FGFR1 | 613 | 38.945 | No |  | 1FGK |
| FGFR1 | 653 | 56.675 | Yes |  | 1FGK |
| FGFR1 | 654 | 149.18 | Yes |  | 1FGK |
| FGFR1 | 677 | 48.29 | Yes |  | 1FGK |
| FGFR1 | 701 | 24.435 | Yes |  | 1FGK |
| FGFR1 | 730 | 32.055 | Yes |  | 1FGK |
| IRS-1 | 18 | 83.615 | No |  | 1QQG |
| IRS-1 | 46 | 17.59 | Yes |  | 1QQG |
| IRS-1 | 47 | 12.435 | No |  | 1QQG |
| IRS-1 | 87 | 5.06 | No |  | 1QQG |
| IRS-1 | 107 | 42.02 | No |  | 1QQG |
| IRS-1 | 183 | 11.48 | No |  | 1QQG |
| IRS-1 | 183 | 18.4 | No |  | 1IRS |
| IGFR-1 | 980 | 32.39 | Yes |  | 1P4O |
| IGFR-1 | 987 | 36.21 | No |  | 1P4O |
| IGFR-1 | 1014 | 54.825 | No |  | 1P4O |
| IGFR-1 | 1090 | 43.675 | No |  | 1P4O |
| IGFR-1 | 1125 | 7.625 | No |  | 1P4O |
| IGFR-1 | 1161 | 7.88 | Yes | Activation Loop, Exposed Upon Phosphorylation | 1P4O |
| IGFR-1 | 1165 | 42.115 | Yes | Activation Loop | 1P4O |
| IGFR-1 | 1166 | 53.835 | Yes | Activation Loop | 1P4O |
| IGFR-1 | 1192 | 75.195 | No |  | 1P4O |
| IGFR-1 | 1213 | 17.275 | No |  | 1P4O |
| IGFR-1 | 1251 | 93.83 | No |  | 1P4O |
| IGFR-1 | 1280 | 31.345 | Yes |  | 1P4O |
| IGFR-1 | 1281 | 65.13 | Yes |  | 1P4O |
| InsR | 941 | 16.74 |  |  | 2DTG |
| InsR | 943 | 0 |  |  | 2DTG |
| InsR | 947 | 208.53 |  |  | 2DTG |
| InsR | 1038 | 56.63 | No |  | 1IRK |
| InsR | 1114 | 12.9 | No |  | 1IRK |
| InsR | 1149 | 2.15 | Yes |  | 1IRK |
| InsR | 1185 | 32.47 | Yes | Activation Loop | 1IRK |
| InsR | 1189 | 26.16 | Yes | Activation Loop | 1IRK |
| InsR | 1190 | 49.72 | Yes | Activation Loop | 1IRK |
| InsR | 1237 | 19.72 | No |  | 1IRK |
| InsR | 1254 | 88.68 | No |  | 1IRK |

**Table S5. Expression of SH2 domains within Muscle and Pancreas.**

Tissue expression data from Unigene was used to determine the expression of specific SH2 domains in either muscle or pancreas. The method for defining SH2 domain families is available on [http://www.sh2domain.org](http://www.sh2domain.org/) [3]. N.E.D. – No expression detected

| **Family** | **Human Gene Names** | **Expression in Muscle** | **Expression in Pancreas** |
| --- | --- | --- | --- |
| ABL | Abl1, Abl2 | Abl1, Abl2 | Abl1 |
| CBL | Cbl, CblB, CblC | Cbl, CblB | Cbl, CblB, CblC |
| CHN | Chn1, Chn2 | Chn1 | Chn1 |
| CRK | Crk, CrkL | CrkL | Crk, CrkL |
| CSK | Csk, Matk | N.E.D. | Csk, Matk |
| DAPP1 | Dapp1 | Dapp1 | N.E.D. |
| FPS | Fes, Fer | Fes | N.E.D. |
| FRK | Frk, Brk, Srms | N.E.D. | N.E.D. |
| GRB2 | Grb2, Gads, Grap | Grb2 | Grb2 |
| GRB7 | Grb7, Grb10, Grb14 | Grb10 | Grb7, Grb10, Grb14 |
| JAK | Tyk2, Jak1, Jak2, Jak3 | Tyk2, Jak1, Jak2 | Tyk2, Jak1, Jak2 |
| NCK | Nck1, Nck2 | Nck1 | Nck1, Nck2 |
| PI3KR | Pik3r1, Pik3r2, Pik3r3 | Pik3r2, Pik3r3 | Pik3r2, Pik3r3 |
| PLCγ | Plcg1, Plcg2 | Plcg1 | N.E.D. |
| PTPN | Ptpn6, Ptpn11 | Ptpn6 | Ptpn6 |
| RASA1 | Rasa1 | Rasa1 | Rasa1 |
| RIN | Rin1, Rin2, Rin3 | Rin2 | Rin1, Rin2 |
| SH2B | Aps, Lnk, Sh2b | Aps, Lnk, Sh2b | Lnk, Sh2b |
| SH2D1 | Sh2d1a, Sh2d1b | N.E.D. | N.E.D. |
| SH2D2 | Sh2d2a, Hsh2, Sh2d7 | N.E.D. | Sh2d2a |
| SH2D3 | Sh2d3a, Sh2d3c, Bcar3 | Sh2d3c | Sh2d3a, Bcar3 |
| SH2D4 | Sh2d4a, Sh2d4b | Sh2d4a | N.E.D. |
| SH2D5 | Sh2d5 | N.E.D. | N.E.D. |
| SH3BP2 | Sh3bp2 | Sh3bp2 | N.E.D. |
| SHB | Shb, Shd, She, Shf | Shb, Shd, She | Shb |
| SHC | Shc1, Shc2, Shc3, Shc4 | Shc1 | Shc1, Shc2 |
| SHIP | Ship1, Ship2 | Ship2 | Ship2 |
| SLAP | Slap, Slap2 | N.E.D. | Slap |
| SLP76 | Slnk, Blnk, Slp76, Mist | Mist | Blnk, Slp76 |
| SOCS | Socs1, Socs2, Socs3, Socs4, Socs5, Socs6, Socs7, Cish | Socs1, Socs4, Socs6 | Socs1, Socs2, Socs3, Socs4, Socs5 |
| SRC | Src, Fyn, Lck, Fgr, Yes, Lyn, Hck, Blk | Src, Fyn, Fgr, Yes, Lyn | Src, Fyn, Lck, Yes, Lyn, Hck, Blk |
| STAP | Bks, Brdg1 | N.E.D. | Bks |
| STAT | Stat1, Stat2, Stat3, Stat4, Stat5a, Stat5b, Stat6 | Stat1, Stat2, Stat3, Stat4, Stat5a, Stat5b, Stat6 | Stat1, Stat2, Stat3, Stat5a, Stat5b, Stat6 |
| SPT6 | Supt6h | Supt6h | Supt6h |
| SYK | Zap70, Syk | Zap70, Syk | Syk |
| TEC | Bmx, Tec, Btk, Itk, Txk | Tec, Txk | N.E.D. |
| TNS | Tns1, Tns3, Tenc1, Tns4 | Tns1, Tns3, Tenc1 | Tns1, Tns3, Tenc1, Tns4 |
| VAV | Vav1, Vav2, Vav3 | Vav3 | Vav1, Vav2 |

**Supplemental Figures**


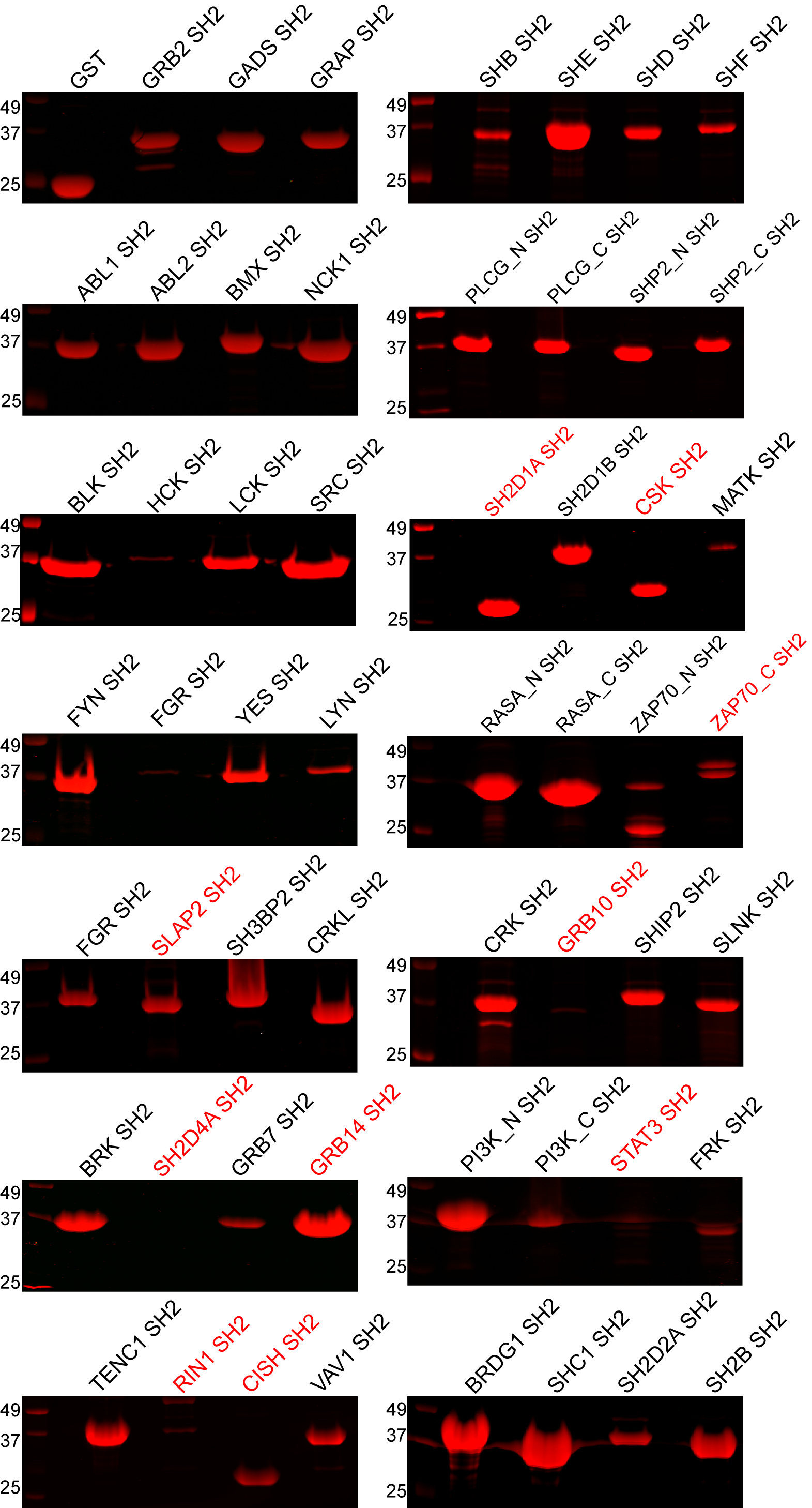


**Figure S1. Expression of GST-SH2 domains used in this study**.

Soluble GST-SH2 constructs are shown on colloidal stained SDS-page gels. Expression of the 50 SH2 domains in this study and other SH2 domains are indicated. See Table S2 for details on the boundaries, constructs and expression of the SH2 domains tested.


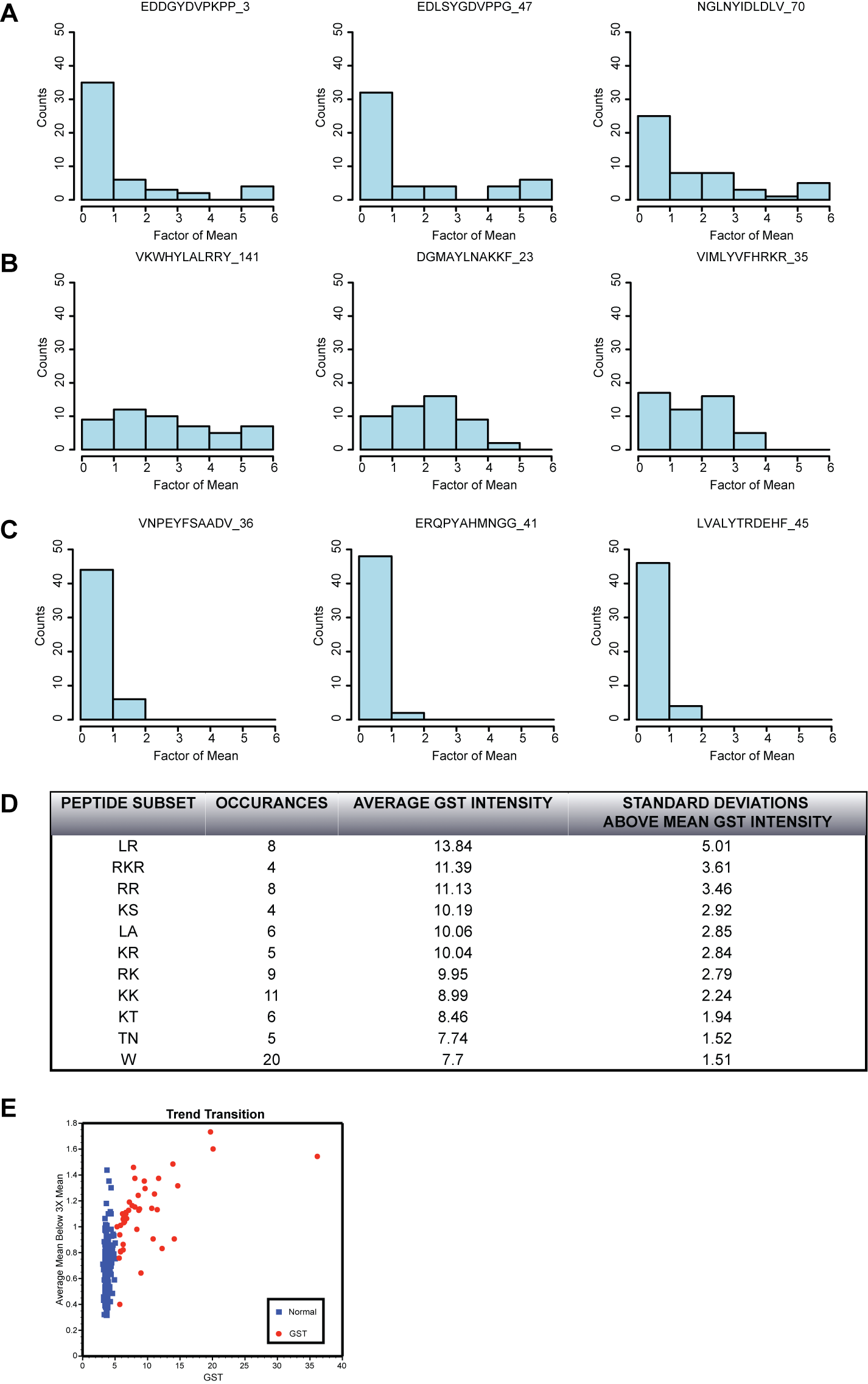


**Figure S2. GST background analysis**

Measured intensity patterns reveal specific, non-specific, and non-binding peptides. The

average binding intensity values for each array probed by an SH2 domain was calculated. Measured intensities for each peptide were expressed as a factor of the mean intensity for each array, allowing values to be compared across all arrays. Each peptide was then analyzed for its binding intensities across all SH2 domains and identified as falling into one of three categories. Histograms indicate the number of interactions scored as a function of the factor of mean intensities. (A) Specific interacting peptides display a bimodal distribution with a significant number of sub-mean intensity values as well as a number of interactions greater than three times mean intensity. (B) Peptides displaying non-specific interactions tend to shift higher in mean intensities. These were confirmed as binding GST with above mean intensity in two out of three independent experiments. (C) Non-interacting peptides have consistently low binding with signals at or below the mean across the majority of experiments. (D) The general trend for poly-basic (Lys or Arg) peptides result in a increase in GST binding. (E) Two trends exist in this analysis. The first trend has a narrow distribution in the low end of the GST affinity intensity and a broad spectrum in the average of the peptide distribution. This grouping constitutes peptides with negligible GST ‘stickiness’, and we regard the affinity results from these peptides to be negligibly effected by the GST in the SH2-GST fusion proteins. The second trend has a broad distribution in the middle to upper range of GST affinity intensity. There exists also a broad distribution in the average of peptides, though heavily weighted to the upper end. This grouping constitutes peptides with crippling GST ‘stickiness’, and we regard the affinity results from these peptides to be unacceptably affected by GST in the SH2-GST fusion proteins. The transition between the aforementioned trends occurs approximately at the mean GST affinity intensity for all three GST trials.

(A)

**
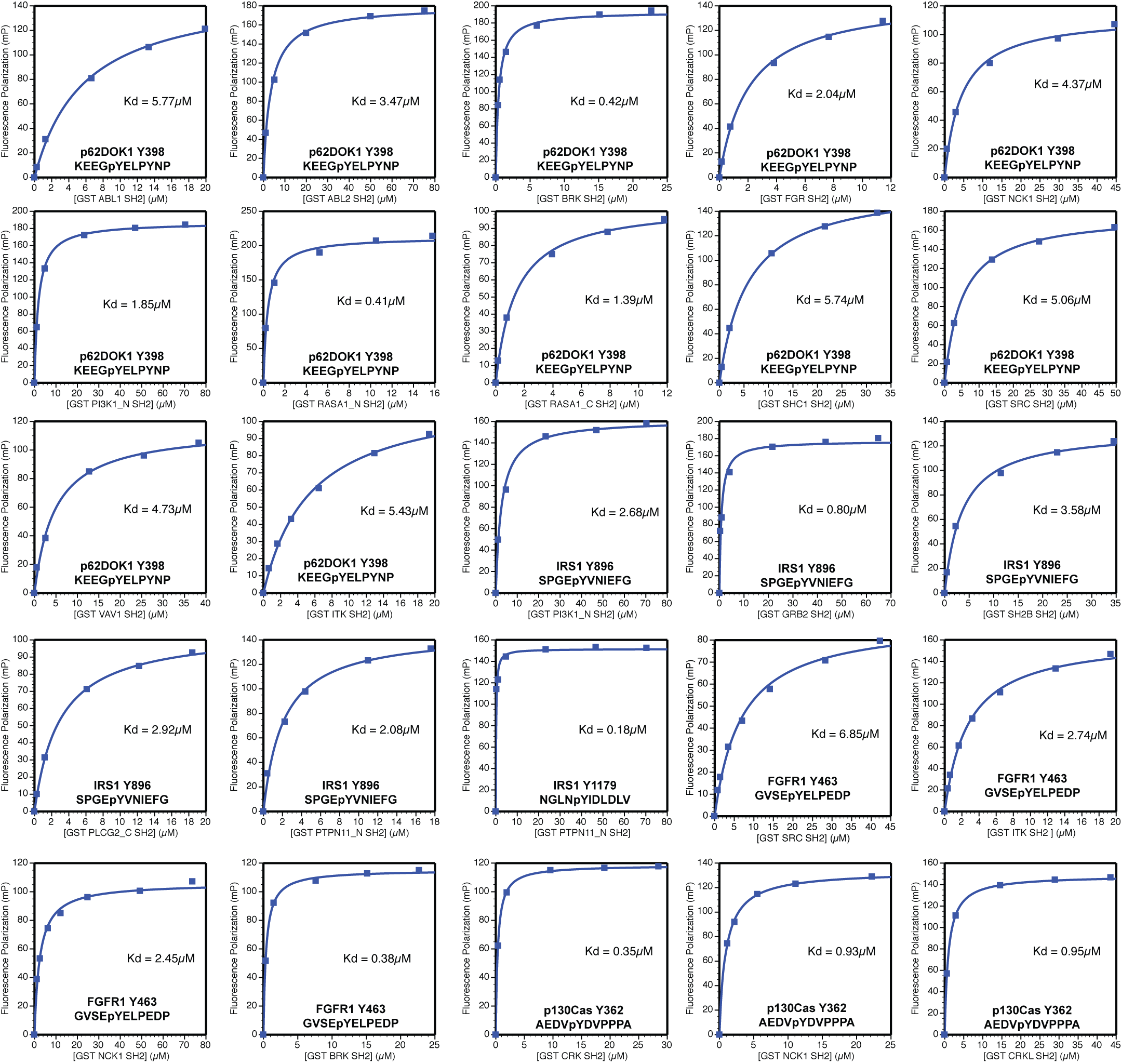
**

**(B)**

**
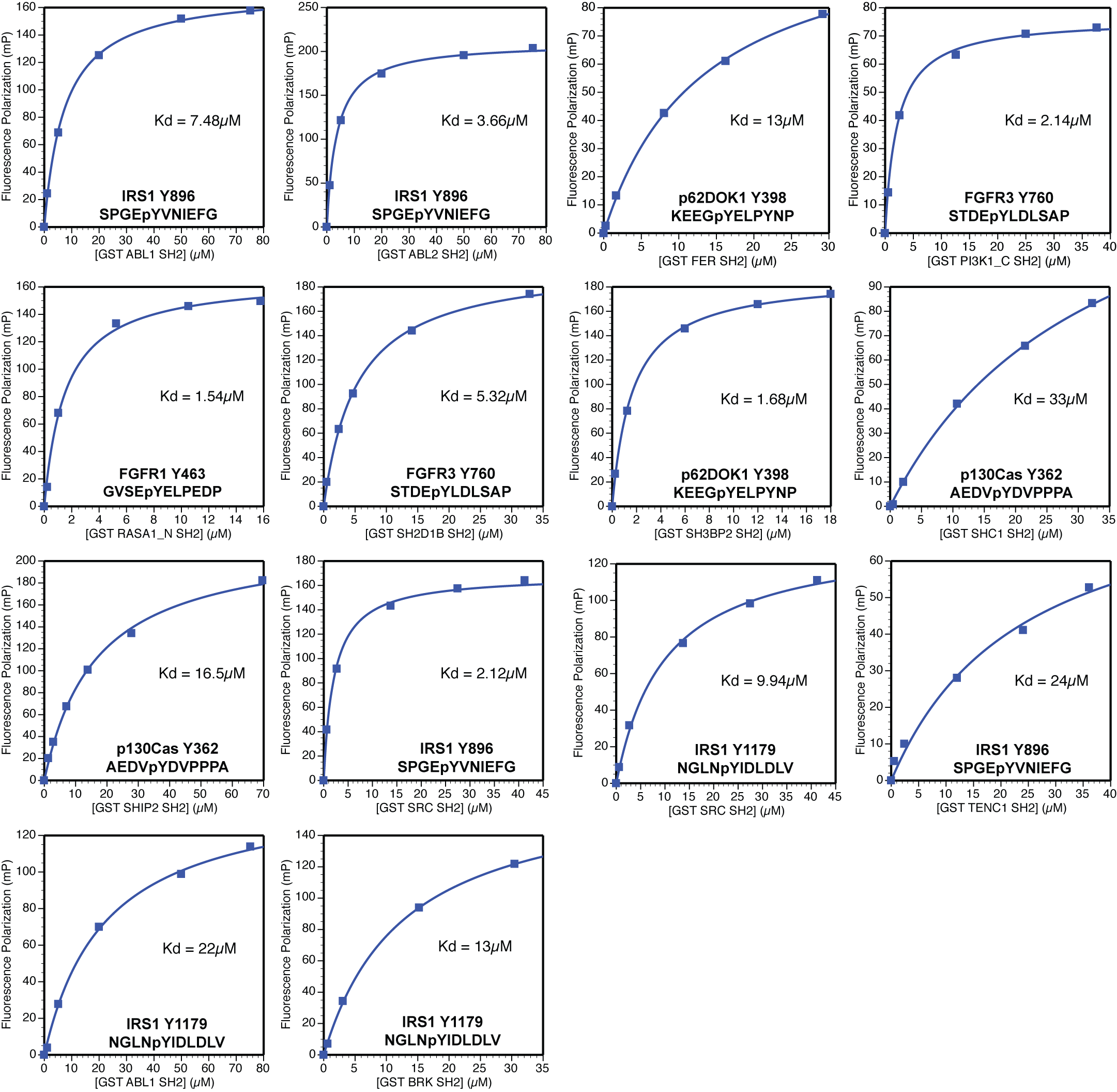
**

**(C)**

**
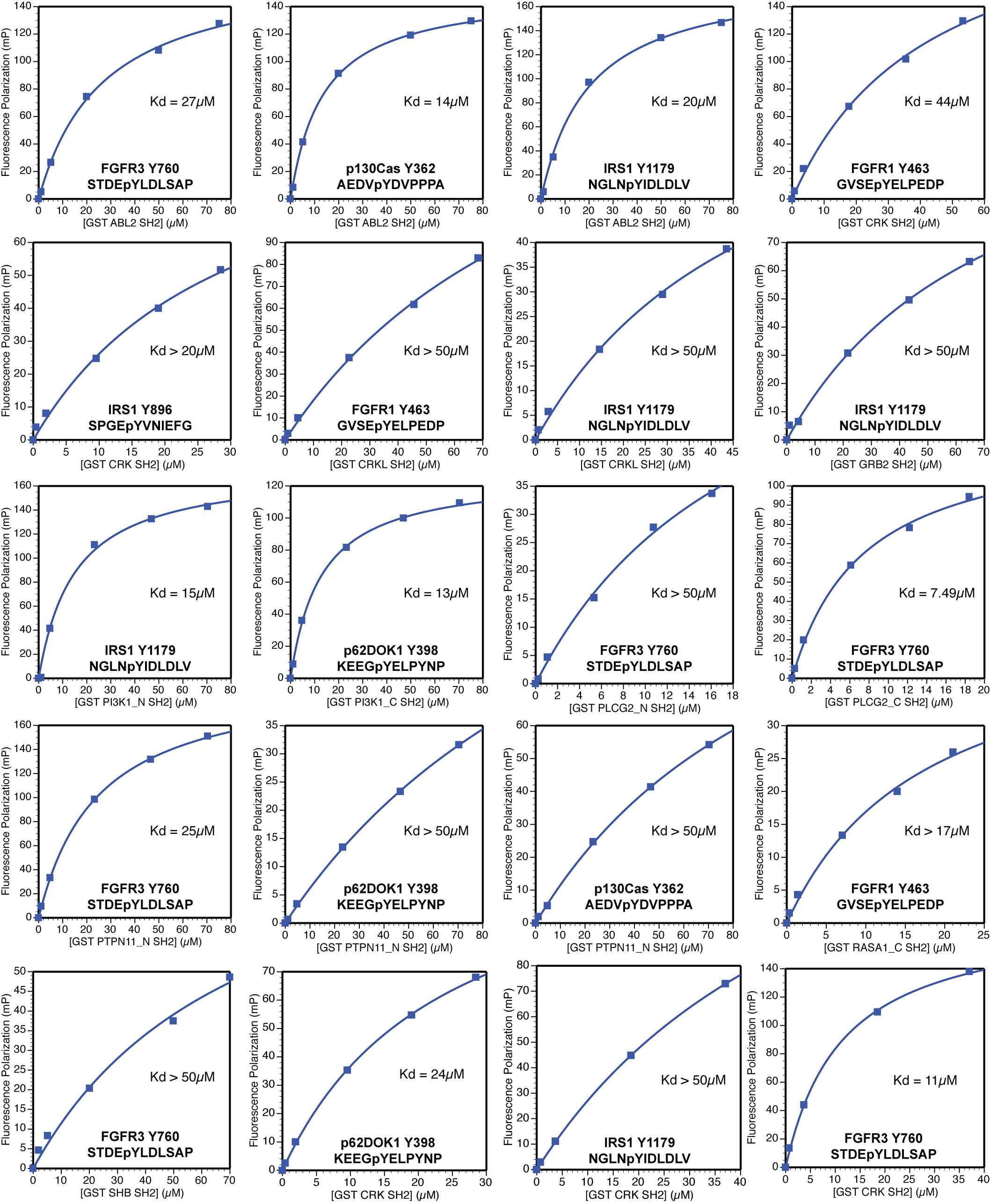
**

**Figure S3. Fluorescence polarization binding curves for 55 SH2 domain and peptide pairs**

Binding Affinities determined by Fluorescence Polarization. Rhodamine B-labeled peptides were mixed with varying concentrations of GST-tagged SH2 domains. Fluorescence polarization measurements were read on a Beacon 2000 (Invitrogen). The curves were fit to a Michaelis-Menten equation to calculate the dissociation constant, K_D_. (A) Peptide binding affinities for interactions greater than 3X the mean (B) Binding affinities of interactions between 1X and 3X the mean intensity on the SPOT array. (C) Affinity measurements that fell below the 2X mean intensity and were considered array negative on the SPOT array.

**
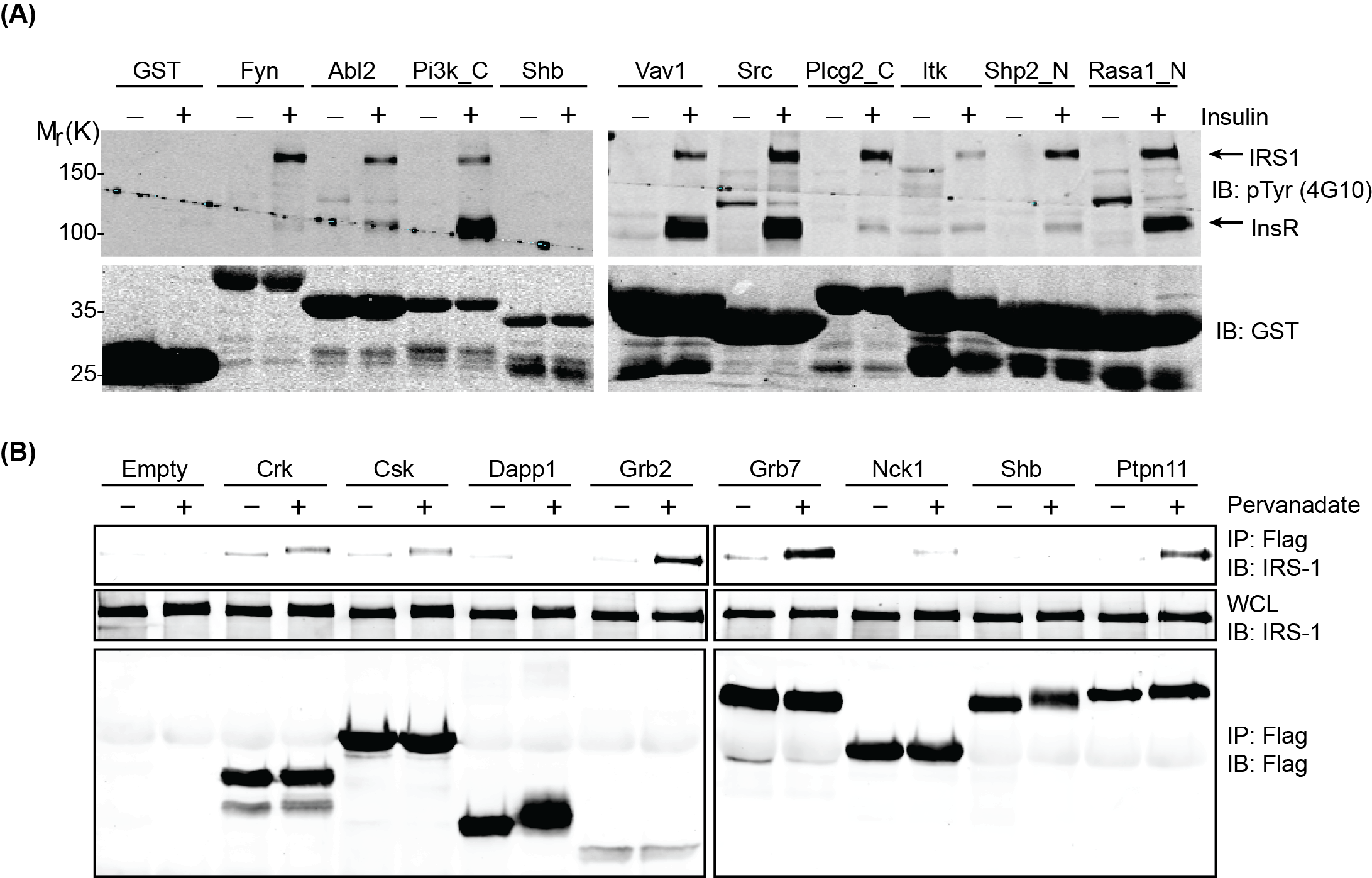
**

**Figure S4. SH2 domain interactions with activated InsR or IRS-1.**

(A) SH2 domains interact with phospho-IRS1 and phosphor_InsR following insulin stimulation. GST-SH2 domains precipitated tyrosine phosphorylated insulin receptor (InsR) and IRS-1 in CHO-IR/IRS1 stably expressing cells upon stimulation with and/or without insulin (100nM) for 5 mins. Tyrosine phosphorylated InsR and IRS-1 were detected using anti-phosphotyrosine antisera (4G10). (B) 3xFlag full length SH2 constructs were transfected into CHO-IR/IRS1 cells for 36 hours and stimulated with pervanadate for 15 mins. Immunoprecipitation using Flag agarose beads reveal interactions with activated IRS-1.


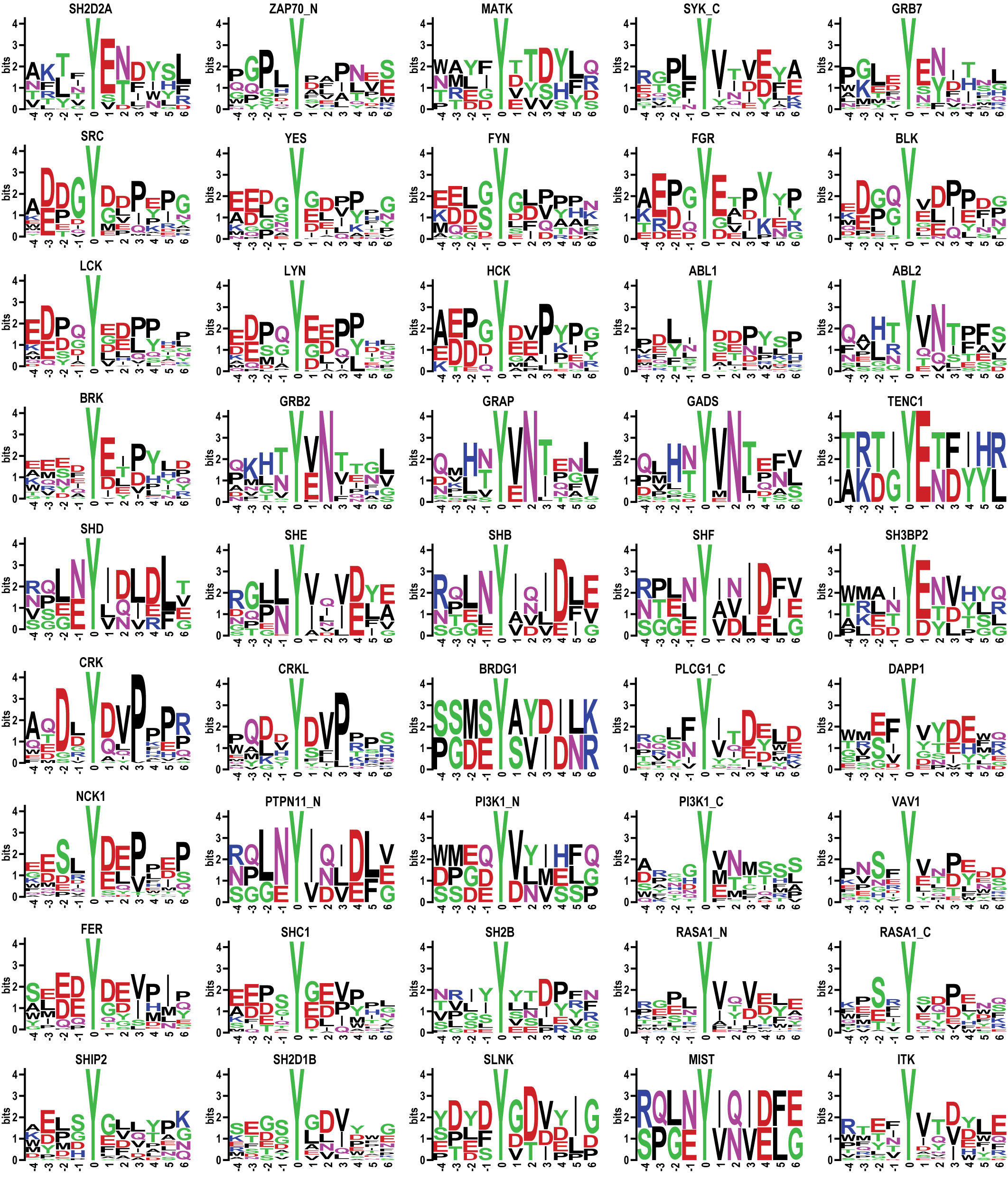


**Figure S5. Physiological ligand specificity for SH2 domains represented as EDSM logos**

Logos for 45 SH2 domains reveal extended specificity for physiological ligands. For each SH2 domain, the array positives (peptides with intensities greater than 3X the mean) were used to generate a represented EDSM logo using WebLogo (http://weblogo.berkeley.edu)[4]. Five SH2 domains were excluded because fewer than two peptides had intensities greater than 3X the mean.


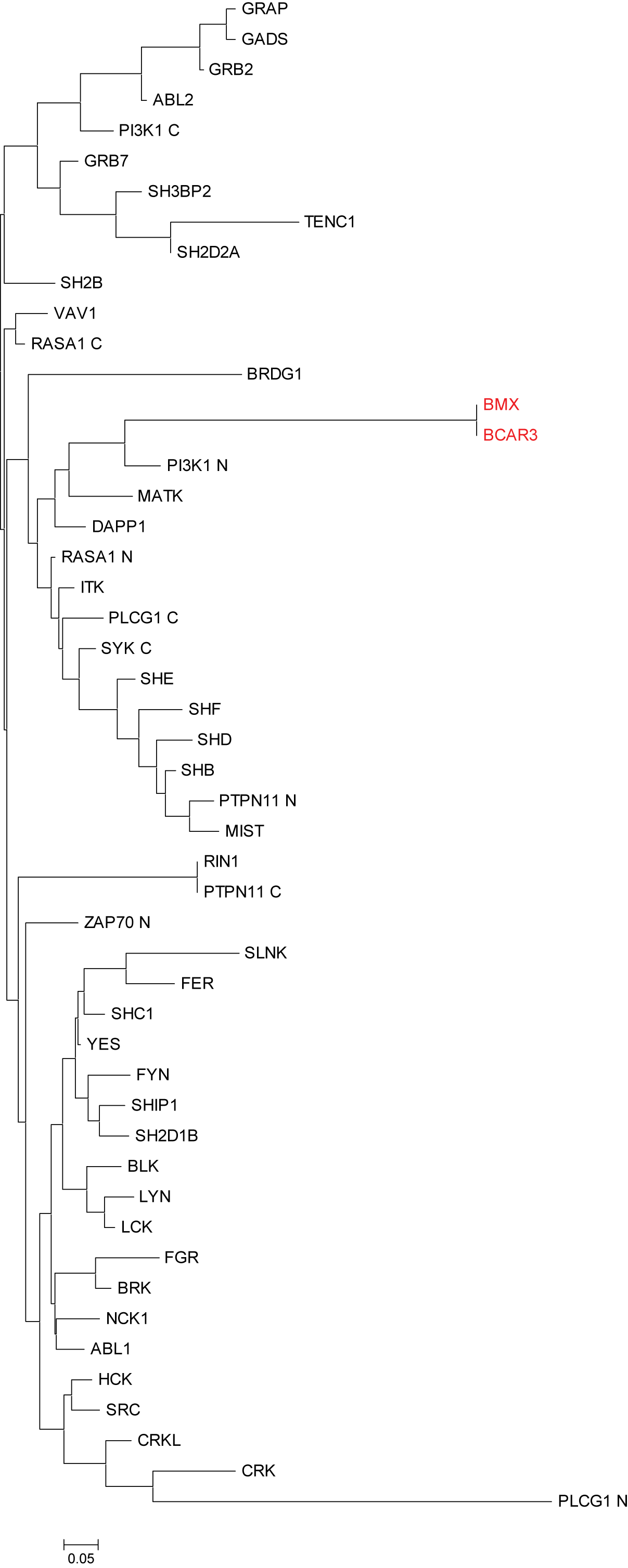


**Figure S6. EDSM Clustering**

A dendogram of the EDSM profiles for the 50 SH2 domains tested in this study. Highlighted in red are SH2 domains with fewer than 2 array positive peptides and therefore specificity information is uninformative.


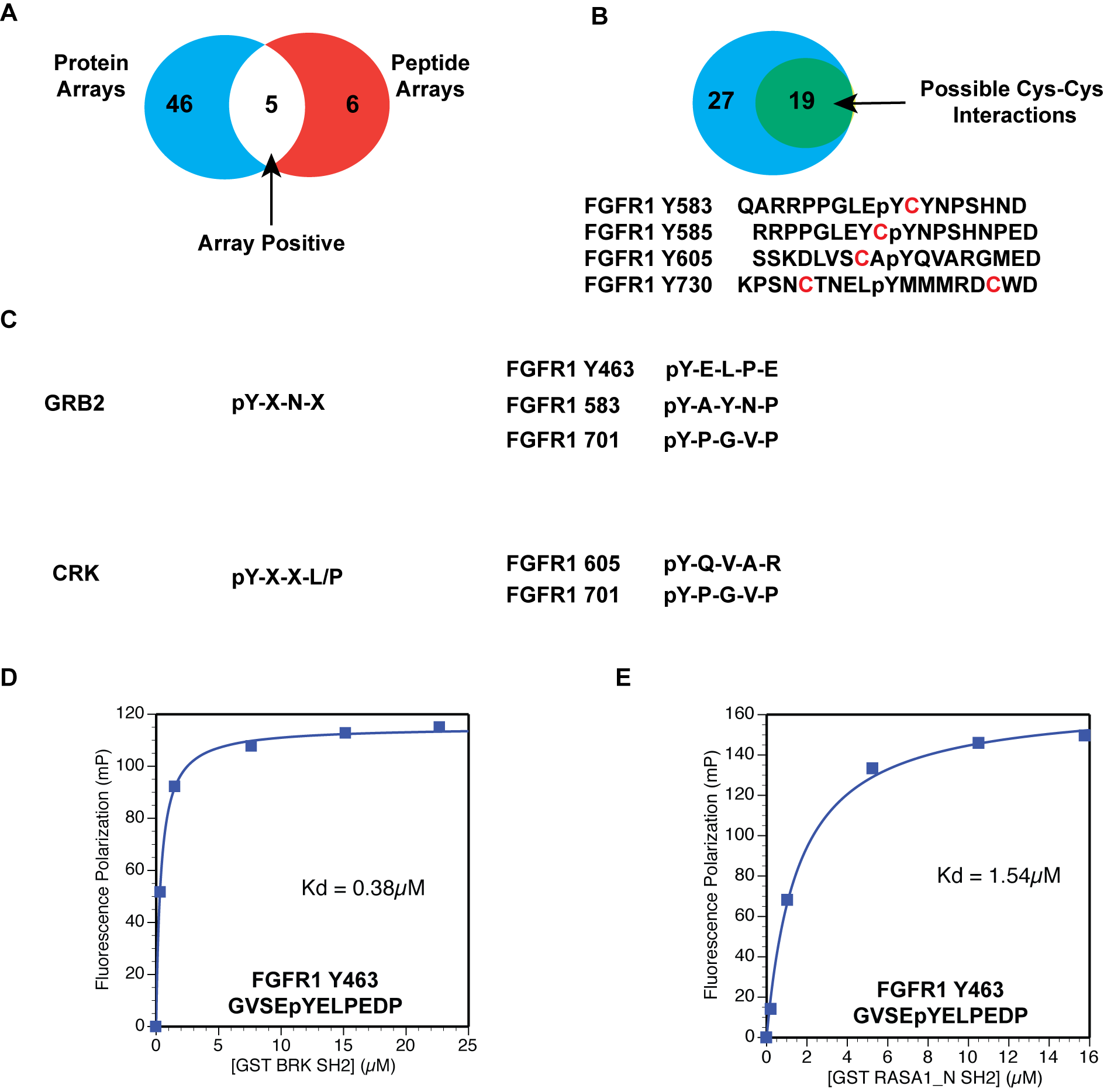


**Figure S7. Comparison between peptide arrays and SH2 protein microarrays**

(A) Venn diagram comparing the positive binders between protein microarrays from Kaushansky et al (< 2µM) [5] and peptide arrays (>3x mean) using overlapping peptides of FGFR1 and select SH2 domains. (B) Of the 46 interactions that were not array positive on peptide arrays, 19 of them were potential disulfide link interactions with specific Cys-containing peptides (listed below) with Cys-containing SH2 domains. (C) Protein arrays detect peptides that do not contain the major secondary factors for GRB2 (+2 Asn) and CRK (+3 Leu/Pro) [6, 7]. (D) The binding affinity (K_D_) for BRK and FGFR1 Y463 confirms array positives using peptide arrays while not detected on protein arrays. (E) In solution binding by fluorescence polarization does not correlate with affinity measurements determined by protein arrays (175nM).

**References**

1. Scheffer T, Wrobel S: **Finding the most interesting patterns in a database quickly by using sequential sampling.** *Journal of Machine Learning Research* 2002, **3:**833-862.

2. Agrawal R, Imielinski T, Swami A: **Mining association rules between sets of items in large databases.** In *1993 ACM SIGMOD International Conference on Management of Data*. Edited by Jajodia PBaS. ACM Press; 1993: 207–216.

3. Liu BA, Shah E, Jablonowski K, Stergachis A, Engelmann B, Nash PD: **The SH2 Domain-Containing Proteins in 21 Species Establish the Provenance and Scope of Phosphotyrosine Signaling in Eukaryotes.** *Sci Signal* 2011, **4:**ra83.

4. Crooks GE, Hon G, Chandonia JM, Brenner SE: **WebLogo: a sequence logo generator.** *Genome Res* 2004, **14:**1188-1190.

5. Kaushansky A, Gordus A, Chang B, Rush J, MacBeath G: **A quantitative study of the recruitment potential of all intracellular tyrosine residues on EGFR, FGFR1 and IGF1R.** *Mol Biosyst* 2008, **4:**643-653.

6. Songyang Z, Shoelson SE, Chaudhuri M, Gish G, Pawson T, Haser WG, King F, Roberts T, Ratnofsky S, Lechleider RJ, et al.: **SH2 domains recognize specific phosphopeptide sequences.** *Cell* 1993, **72:**767-778.

7. Liu BA, Jablonowski K, Shah EE, Engelmann BW, Jones RB, Nash PD: **SH2 domains recognize contextual peptide sequence information to determine selectivity.** *Mol Cell Proteomics* 2010, **9:**2391-2404.
